# Size Switchable Supramolecular Nanoparticle Based on Azobenzene Derivative within Anionic Pillar[5]arene

**DOI:** 10.1038/srep37014

**Published:** 2016-11-16

**Authors:** Cai-Cai Zhang, Sheng-Hua Li, Cui-Fang Zhang, Yu Liu

**Affiliations:** 1 Department of Chemistry, State Key Laboratory of Elemento-Organic Chemistry, Nankai University, Tianjin 300071, P. R. China; 2 Collaborative Innovation Center of Chemical Science and Engineering (Tianjin), Nankai University, Tianjin 300071, P. R. China

## Abstract

A photo/thermal-switchable supramolecular nanoparticles assembly has been constructed based on an inclusion complex between anionic pillar[5]arene **2C-WP5A** and azobenzene derivative **Azo-py-OMe (G)**. The novel anionic pillar[5]arene-based host-guest inclusion complexation was investigated by the ^1^H NMR titration, 2D ROESY and isothermal titration microcalorimetry (ITC) showing high association constant (*K*_a_) of (2.60 ± 0.06) × 10^4^ M^−1^ with 1:1 binding stoichiometry. Furthermore, the supramolecular nanoparticles assembly can be conveniently obtained from **G** and a small amount of **2C-WP5A** in aqueous solution, which was so-called “host induced aggregating (**HIA**)”. The size and morphology of the supramolecular nanoparticles assembly were characterized by TEM and DLS. As a result of the photo/thermal-isomerization of **G** included in the cavity of **2C-WP5A**, the size of these nanoparticles could reversibly change from ~800 nm to ~250 nm, which could switch the solution of this assembly from turbid to clear.

Recently, size switchable materials have attracted much attention, as size is the fundamental and crucial factor that determined the properties of materials, for example as the noble metal nanoparticles^1^, polymeric microparticles^2^ or colloidal superparticles^3^. The stimuli-responsive molecules might be very useful to build up the size switchable systems^4^,^5^. Azobenzene is one of the most attractive stimuli-responsive molecules^6^,^7^, because of its reversible isomerization between *trans* and *cis* conformation upon external stimuli, such as mechanical stress^8^, electrostatic stimulation^9^,^10^, as well as light irradiation^11^,^12^,^13^. So the supramolecular assemblies involved azobenzene have various applications in intelligent membranes^14^,^15^, optical memories^16^,^17^,^18^ and biological systems^19^,^20^,^21^ and so on. A large number of photo-responsive systems based on the azobenzene and macrocycles have been reported^22^,^23^,^24^,^25^,^26^, such as, a photo regulable ion-extraction and ion-transport based on azobenzene-crown ethers^27^, a photo-responsive artificial muscle based on host-guest complex of α-cyclodextrin and azobenzene^28^,^29^, and a dual stimuli-responsive self-assembly supramolecular nanoparticles based on the ternary host-guest complexation between cucurbit[8]uril, a methyl viologen polymer, and mono- and multivalent azobenzene functionalized molecules^30^.

As a new class of macromolecules, pillar[n]arenes (5–15) have been extensively studied from their synthesis, functionalization and supramolecular chemistry since 2008^31^,^32^,^33^,^34^,^35^,^36^. The first photo-responsive host-guest complexation based on pillar[6]arene and azobenzene-guest was reported by Huang in 2012^37^. Thereafter, the author constructed a dual-responsive supra-amphiphilic polypseudo-rotaxane based on pillar[7]arene and azobenzene derivative in aqueous solution^38^. Meanwhile, Ogoshi also reported a photoreversible switching of the lower critical solution temperature (LCST) in host-guest system of pillar[6]arene and azobenzene derivative^39^. Up to now, most reported photo-responsive systems based on azobenzene and pillar[n]arene are mainly focus on pillar[6,7]arene^40^,^41^,^42^. Nevertheless, the diameter of the internal cavity of pillar[5]arene is ~4.7 Å, which is equal to α-cyclodextrin (~4.7 Å)^43^. In accordance with common sense, it is reasonable that the pillar[5]arene and azobenezene derivative could form host-guest inclusion complexation. Ogoshi has studied the threading process of pillar[5]arene onto a viologen derivative containing an azobenzene end-group^44^. But there has been no direct proof to reveal the host-guest complexation between anionic pillar[5]arene and photo-responsive azobenzene derivatives. Considering the easier access to pillar[5]arene compared with pillar[6,7]arene and the aforementioned reasons, it is necessary to investigate the host-guest complexation and assembly behavior based on pillar[5]arene and azobenzene derivatives. In addition, to the best of our knowledge, size switchable supramolecular nanoparticles formed by host-guest interactions between anion pillar[5]arene and azobenzene derivatives have been underexplored.

Herein, we present a feasible strategy for fabricating photo/thermal-responsive size switchable supramolecular nanoparticles assembly based on the unprecedented host-guest inclusion complex between anionic pillar[5]arene **2C-WP5A** and azobenzene derivation **G** in aqueous solution (Fig. 1). The design proposed here combines the following advantages: (1) the percarboxylatopillar[5]arenes is the most used anionic pillar[5]arene, which can be obtained easily and efficiently; (2) differing from the α-cyclodextrin, either *trans-* and *cis-* azobenzene derivatives **G** could be included in **2C-WP5A** with similar affinity, which could result in the only reversible change of the nanoparticle’s size without disassembling; (3) the size change by photo/thermal-stimulation would result in the reversible switching the transparency of solution from turbid to clear.

## Results and Discussion

### Host-Guest Complexation For 2C-WP5A and *trans*-G

**Figure 2:**
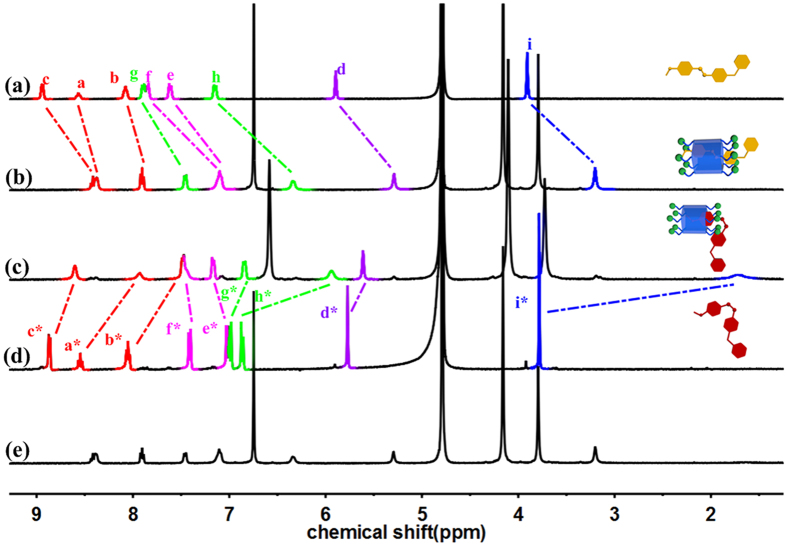
Partial ^1^H NMR spectra (D_2_O, 25 °C, 400 MHz) of (**a**) ***trans-*****G** (1.6 mM); (**b**) ***trans*****-G** (1.6 mM) and **2C-WP5A** (1.6 mM); (**c**) ***trans*****-G** (1.6 mM) and **2C-WP5A** (1.6 mM) after irradiation at 365 nm for 10 min; (**d**) ***trans*****-G** (1.6 mM) after irradiation at 365 nm for 10 min; (**e**) ***trans*****-G** (1.60 mM) and **2C-WP5A** (1.6 mM) after further heating at 80 °C for 1 h.

The host-guest complexation of **2C-WP5A** and ***trans-*****G** was first investigated by ^1^H NMR experiment in D_2_O at 25 °C (Fig. 2b). Signals for the protons on ***trans-*****G** were significantly shifted upfield in the presence of 1 equiv. **2C-WP5A**. This result suggested that the formation of the threaded structure **2C-WP5A**
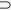
***trans-*****G**, in which these protons of the guest molecule were shielded by the aromatic rings in **2C-WP5A**. The Job’s plot was employed to find the binding stoichiometry of **2C-WP5A**
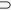
***trans*****-G**, the top point of the curve was appeared at the molar fraction *X*_guest_ = 0.5 (see Supplementary Fig. S12), indicating the 1:1 binding stoichiometry between **2C-WP5A** and **G**.

Interestingly, comparing to the chemical shift of the protons on the pyridinium unit, the signals of the protons on the azobenzene unit changed more obviously. The complexation-induced chemical shifts (Δδ) followed the order (see Supplementary Fig. S13): H_h_ > H_g_ > H_i_ > H_d_ > H_e_ = H_c_ > H_f_ > H_b_ > H_a_. These shifts of methyl and both aromatic rings of the azobenzene unit were shielded by the pillar[5]arene, and the ring A is more shielded than others, which indicated that the aromatic rings of **2C-WP5A** were located at the azobenzene unit of ***trans-*****G** especially at ring A, instead of pyridinium unit. Although we failed to get the single crystal of the **2C-WP5A**
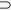
***trans*****-G**, the 2D ROESY NMR experiment (see Supplementary Fig. S14) also supported our speculation of the binding mode. A 2D ROESY spectrum of an equimolar (5 mM) mixture of ***trans*****-G** and **2C-WP5A** in D_2_O was obtained to confirm the peak assignments. NOE correlation signals were observed between protons H_g_, H_e_ and H_f_ on the azobenzene unit of ***trans-*****G** and side chain protons H_3_ and phenyl moieties protons H_2_ on **2C-WP5A**. The results showed that azobenzene unit was indeed threaded in the cavity of anionic **2C-WP5A**, which was consistent with the above-mentioned ^1^H NMR results. To further confirm this result, we synthesized another guest molecule **G′** with 1,4-diazabicyclo[2.2.2]octane group. The additional experiment results (see Supplementary Figs S15 and S16) also supported our conclusion that the azobenzene unit was positioned inside the cavity of **2C-WP5A**. Recently, an inclusion complex of azo dye and cationic pillar[5]arenes was reported by Stoikov, which is also confirmed that azobenzene unit threaded in the cavity of pillar[5]arene^45^.

### The photo/thermal responsive properties of G

To further investigation of the photo-responsive properties of the host-guest complex and supramolecular assembly of **2C-WP5A** and **G**, we firstly examined the photoisomerization behavior of **G** by UV/vis (see Supplementary Fig. S17) and ^1^H NMR (Fig. 2d). The UV/vis spectrum of ***trans*****-G** has a strong absorption maximum at 351 nm, which corresponds to the π-π* transition of the *trans* form. When the solution of ***trans*****-G** was irradiated at 365 nm for 2 min, the main absorption band bleached greatly. Meanwhile, slightly increase in a new band at 432 nm, which is ascribed to the n-π* transition of the *cis* form of the azobenzene. The changes in the UV/vis spectrum indicated that the photoisomerization of **G** from the *trans* to *cis* conformation. Moreover, the *trans/cis* photoisomerization of **G** can be quantitatively calculated by integration of the signals in the ^1^H NMR spectrum. After irradiation with UV light at 365 nm for 10 min to ensure the fully transformed, a series of new signals appeared which is corresponding to the *cis* form, and the ratio of the *trans*:*cis* reached up to 10:90 (Fig. 2d). It is remarkable that **G** has not only photo-responsive but also thermal-responsive performances^46^. The continuous heating of the irradiated solution at 80 °C for one hour resulted in the regeneration of original protons signals of **G**, suggesting the recovery of *trans* form of **G**.

### The photo/thermal responsive properties of 2C-WP5A 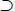 G

After irradiation the solution of 1:1 **2C-WP5A**
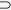
***trans-*****G** with UV light at 365 nm for 10 min, some new signals appeared as shown in Fig. 2c. In comparison to the signals of individual **G** at the same experimental condition (Fig. 2d), we observed that the chemical shift of proton H_i*_ shifted upfield from 3.75 ppm to 1.71 ppm, and the peaks of the protons related to the azobenzene altered slightly (h_e_*, h_f_*, h_g_* and h_h_* Δδ = 0.15, 0.06, −0.13 and −0.92 ppm, respectively). Those changes indicated that the ring B was deshielded by aromatic ring of pillararene, and the methyl group was more shielded than others, which implied the most of azobenzene unit was threaded out of pillararene for **2C-WP5A**
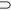
***cis-*****G.** The complexion of **2C-WP5A**
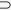
**G** exhibited photo-responsive and thermal-responsive as well, the reversible switchable process of the inclusion complex between **2C-WP5A** and **G** could be modulated upon irradiation with UV at 365 nm and heating at 80 °C for one hour (Fig. 2e).

**Figure 3:**
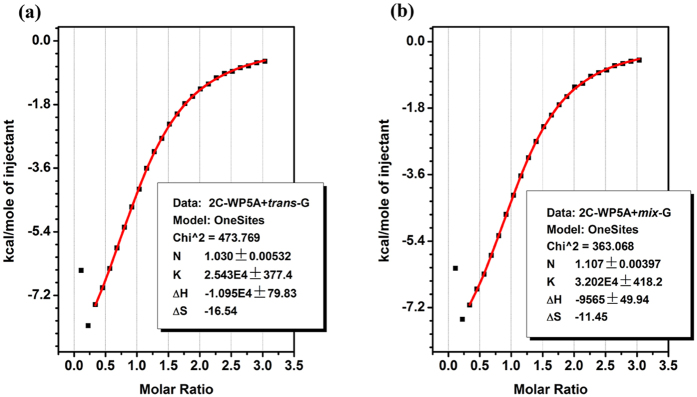
(**a**) Microcalorimetric titration of **2C-WP5A** with ***trans-*****G** in water at 25 °C; (**b**) Microcalorimetric titration of **2C-WP5A** with ***trans-*****G/c*****is-*****G** (molar ratio 10/90) in water at 25 °C. “Net” heat effect obtained by subtracting the heat of dilution from the heat of reaction, which was analyzed by computer simulation using the “one set of binding sites” model.

To further understand the interaction between **2C-WP5A** and *trans/cis-* **G**, we used isothermal titration calorimetry (ITC) to explore the thermodynamics of the host-guest inclusion complex (Fig. 3). The *K*_a_ values and thermodynamic parameters are listed in Table 1, and we are surprised to find that there was no obvious difference between the *K*_a_*-**cis*** and the *K*_a_-***trans***. The hydrophobic interaction is characterized by positive value of ΔH^45^,^47^,^48^, which suggests that the hydrophobic interaction is the main force in the complexation. Meanwhile, **2C-WP5A** and **G** immobilized their conformation during the complexation, resulted in a great negative entropy change which offset the positive entropy change aroused by solvent liberation^49^. This great unfavorable entropy make us easily understand why pillar[5]arene cannot bind with azobenzene in organic solution although its size is similar to α-cyclodextrin.

### Construct supramolecular self-assembly

**Figure 4:**
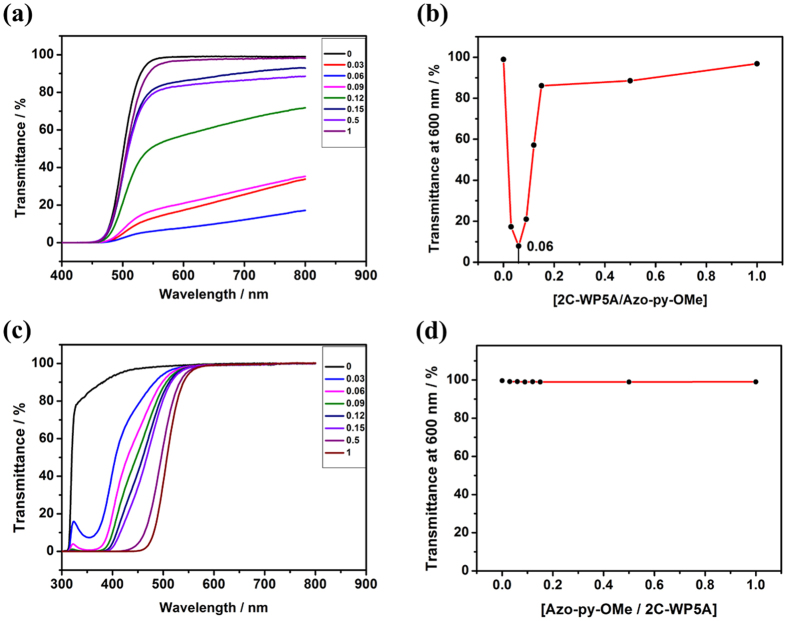
(**a**) Optical transmittance of aqueous solutions of **2C-WP5A** at different concentrations in the presence of ***trans*****-G** (1.6 mM) at 25 °C. (**b**) Dependence of optical transmittance at 600 nm on **2C-WP5A** concentration in the presence of ***trans*****-G** (1.6 mM) at 25 °C. (**c**) Optical transmittance of aqueous solutions of ***trans-*****G** at different concentrations in the presence of **2C-WP5A** (1.6 mM) at 25 °C. (**d**) Dependence of optical transmittance at 600 nm on ***trans-*****G** concentration in the presence of **2C-WP5A** (1.6 mM) at 25 °C.

Recently, we have developed a novel strategy for the construction of supramolecular assemblies by means of host induced aggregating (**HIA**: a small amount of macrocyclic host could promote the aggregation of guest molecules by lowering the critical aggregating concentration (**CAC**) and even regulating the morphology of the aggregates)^50^,^51^,^52^,^53^,^54^,^55^,^56^,^57^. So after successful establishing the recognition motif between **2C-WP5A** and **G** in aqueous solution, we were trying to explore the constructing supramolecular aggregates based on **2C-WP5A** and ***trans-*****G**. **G** could be induced by **2C-WP5A** to form large assembly via **HIA** by monitoring the dependence of the optical transmittance at 600 nm on the concentration of **G** (There is no absorbance of **G** at 600 nm Fig. 4a). It is noteworthy that in the absence of **2C-WP5A**, the optical transmittance of ***trans-*****G** at 600 nm showed no appreciable change as the concentration increased form 0.8 mM to 2.0 mM (see Supplementary Fig. S18). It is requisite to find the best molar ratio between **2C-WP5A** and **G** for fabricating the supramolecular assembly. As shown in Fig. 4b, the optical transmittance at 600 nm of the ***trans-*****G** solution first decreased sharply upon the gradually added **2C-WP5A** until reaching the minimum and then gradually increased as the amount of **2C-WP5A** continued to increase. By monitoring the change of the transmittance at the wavelength of 600 nm in the process, the best molar ratio of ***trans-*****G** and **2C-WP5A** can be determined at 1:0.06. Redundant amount of **2C-WP5A** leads to the formation of a simple inclusion complex and disaggregation of the aggregates, accompanied with the rising of the optical transmittance. This phenomenon highlighted the important role of HIA in the formation of the supramolecular assembly. In fact, only a small amount of macrocyclic host (6%) could promote the aggregation of guest molecules. In order to prove the prominent effect of the macrocyclic structure of host, a control experiment showed that no assembly appeared when replacing **2C-WP5A** with its fragment benzene-1,4-dioxyacetic acid ammonium salt (DAAS) under comparable conditions (see Supplementary Fig. S19), indicating that the cyclic structure of **2C-WP5A** is vital in inducing the supramolecular assembly. Furthermore, in order to make comparison with HIA, we fixed the concentration of **2C-WP5A** at 1.6 mM, gradually added ***trans-*****G** and controlled the experiment procedure same to the HIA. As shown in Fig. 4c,d, the transmittance the **2C-WP5A** is not changed upon the addition of **G** from 0 to 1.0 eq. So for induced aggregating, the poly-charged additive is necessary such as the macrocyclic host molecules. Overall, there are three main points in HIA: (1) strong binding affinity between host and guest; (2) charge interaction between host and guest; (3) the macrocyclic structure of host molecule.

**Figure 5:**
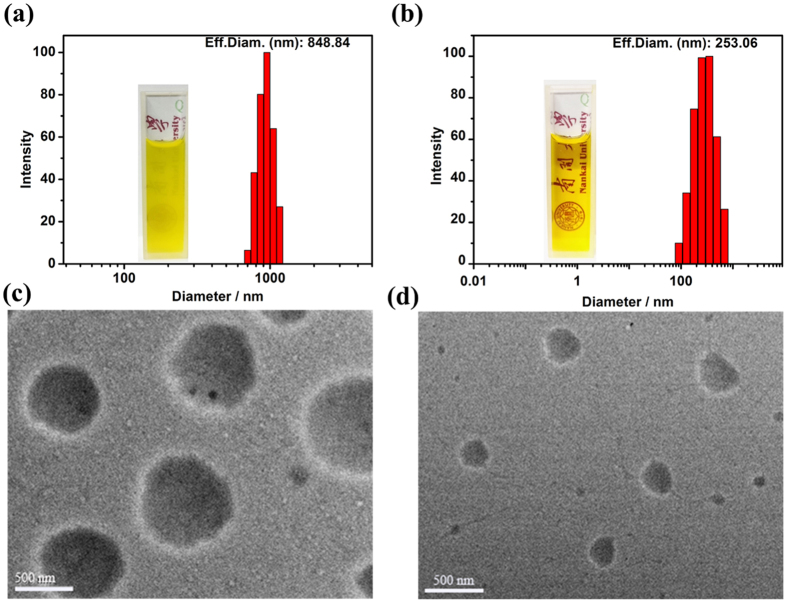
DLS data: (**a**) the **2C-WP5A+*****trans-*****G** assembly at 25 °C. Inset: Photograph of the solution of the **2C-WP5A+*****trans-*****G** assembly. (**b**) After irradiation with UV light at 365 nm of (**a**) for 10 min. Inset: Photograph of the solution of the **2C-WP5A+G** assembly after irradiation with UV light at 365 nm for 10 min. (**c**) TEM image of (**a**). (**d**) TEM image of (**b**).

Dynamic light scattering (DLS) and transmission electron microscopy (TEM) are used to characterize the size and morphology of **2C-WP5A+G** assembly. As shown in Fig. 5a, DLS data showed that **2C-WP5A+*****trans-*****G** formed well-defined aggregates with a narrow size distribution, giving an average diameter about 848 nm at a scattering angle of 90°. Meanwhile, the TEM image (Fig. 5c) also certificated the formation of spherical nanoparticles with diameter about 800 nm consistent with the DLS data. Furthermore, after irradiation the solution with UV light at 365 nm for 10 min, the solution became clear and the size of the aggregates formed by **2C-WP5A+*****cis-*****G** changed into smaller nanoparticles with diameter about 253 nm (Fig. 5b,d). Afterwards, upon heating the solution at 80 °C for one hour, the size of the aggregates returned to the original status. So it is incontrovertible that the isomerization of **G** by photo/thermal stimulation has significant influence on the size and morphology of the supramolecular nanoparticles assembly.

Zeta potential measurement was further performed to identify the supramolecular assembly surface charged distribution (see Supplementary Figs S20 and S21), both of the **2C-WP5A+***trans/cis* **G** assembly giving an average negative zeta potential to maintain the stability of the assembly. Based on the above experimental results and analysis, we postulate that the formation of the supramolecular assembly occurred in two steps. Firstly, the **2C-WP5A** and **G** instantaneously formed a host-guest complex. Subsequently, excess guest molecules arranged around the host-guest complex due to electrostatic effect with redundant carboxylate anion of **2C-WP5A**. According to the experimental results of the zeta potential, the **2C-WP5A** was on the surfaces of the nanoparticles. Based on the above description, the poly-anionic structure of **2C-WP5A** is very important in the formation of the supramolecular nanoparticles assembly.

**Figure 6:**
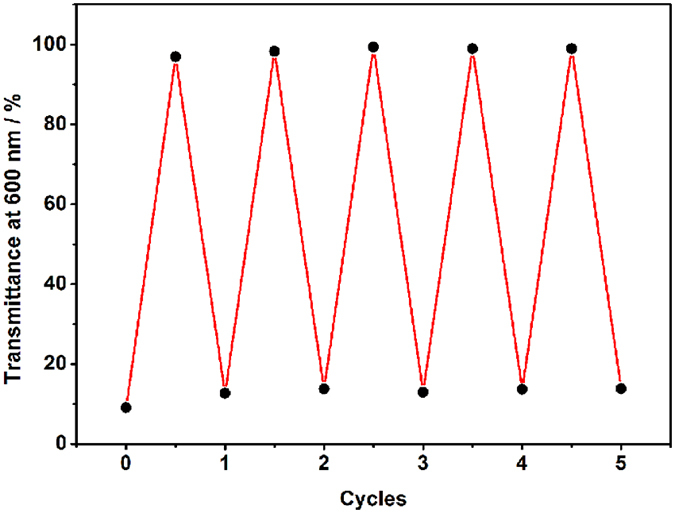
Optical transmittance at 600 nm of 2C-WP5A+G solution observed upon several cycles under irradiation at 365 nm and heating at 80 °C for one hour.

It is also required to make a thorough inquiry about the repetitiveness of the photo/thermal driven size switching of the supramolecular assembly. Size switching process was determined by monitoring the optical transmittance of **2C-WP5A+G** solution at 600 nm (Fig. 6) and DLS experiment (see Supplementary Fig. S22). The presence of the large aggregates of the solution was evident from the decrease in transmittance, which was corresponding to the formation of spherical nanoparticles with diameter of 800 nm from DLS and TEM results. After irradiation with UV light at 365 nm for 10 min, the solution turned clear and transparent with nearly 100% transmittance. By continuous heating at 80 °C for one hour, the solution reverted to the initial turbidity state. Significantly, the reversible transition between turbidity and clarity could repeat several times, indicating that the good photo/thermal responsiveness of the supramolecular assembly.

## Conclusions

In conclusion, a novel recognition motif between anionic pillar[5]arene **(2C-WP5A)** and azobenzene derivative **G** has been developed, and both of the *trans-* and *cis-* **G** have the similar affinity with **2C-WP5A**. The complexation process was well studied and confirmed by ^1^H NMR, 2D ROESY, and ITC experiment. The recognition motif would greatly expand the application in the area of construction of stimuli-responsive supramolecular assembly based on anionic pillar[5]arene. Furthermore, we utilized this novel recognition motif to construct supramolecular assembly nanoparticles which could reversibly switch the size from ~800 nm to ~250 nm by photo/thermal stimulation, presenting the turbid-to-clear switch of the solution state with excellent reversibility. To the best of our knowledge, this is the first example of supramolecular aggregate based anionic pillar[5]arene and azobenzene derivative. The high affinity of anionic pillar[5]arene with azobenzene derivative is expected to have great potential applications in further fabrication of more sophisticated stimuli-responsive supramolecular system.

## Methods

### Materials

**2C-WP5A** was prepared according to the published procedures^58^. All reagents were commercially available and used without further purification, unless otherwise noted. Solvent were dried according to procedures described in the literature. Column chromatography was performed on silica gel (200–300 mesh).

### Measurements

All experiments were performed in deionized water at 25 °C unless noted otherwise.

### NMR Spectra

^1^H NMR and ^13^C NMR spectra were recorded on a Bruker AVANCE AV400 (400 and 100 MHz). Signal positions were reported in part per million (ppm) relative to the residual solvent peaks or to the peak of Si(CH_3_)_4_ used as an internal standard with the abbreviations s, d, t, q, and m, denoting singlet, doublet, triplet, quartlet and multiplet, respectively. The residual ^1^H peak of deuterated solvent appeared at 4.79 ppm in D_2_O, at 7.26 ppm in CDCl_3_. All coupling constants *J* are quoted in Hz.

### High resolution mass spectra (HRMS)

HRMS were performed on an Agilent 6520 Q-TOF LC/MS with ESI ionization.

### DLS Measurement

The sample solution was filtered through a 450 nm Millipore filter into a clean scintillation vial and then was examined using a laser light scattering spectrometer equipped with a digital correlator at 640 nm at a scattering angle of 90°.

### UV/Vis spectra

UV/Vis spectra were recorded in a quartz cell (light path 1 mm) on a Thermo Scientific EVOLUTION 300 spectrophotometer equipped with a HAAKE SC 100 temperature controller to keep the temperature at 25 °C.

### Optical transmittance Measurement

The optical transmittance of the aqueous solution was measured in a quartz cell (light path 10 mm) on a Shimadzu UV-3600 spectrophotometer equipped with a PTC-348WI temperature controller.

### TEM Measurement

The sample for TEM measurement was prepared by dropping the solution onto a copper grid. The grid was then air-dried. The samples were examined by a high-resolution TEM (Tecnai G2 F20 microscope, FEI) equipped with a CCD camera (Orius 832, Gatan) operating at an accelerating voltage of 200 kV.

### ITC experiment

The ITC experiment was carried out at 25 °C in aqueous solution. In a typical experiment, the solution of 2C-WP5A in a 0.250 mL syringe was sequentially injected with stirring at 300 rpm into a solution of G in the sample cell (1.4227 mL). A control experiment to determine the heat of dilution was carried out by performing the same number of injections with the same concentration of host compound into ultrapure water. The dilution enthalpies determined in control experiments were subtracted from the enthalpies measured in the titration experiments to obtain the net reaction heat. All thermodynamic parameters reported in this work were obtained by using the “one set of binding sites” model. Two titration experiments were independently performed to give the averaged values with standard error (see Supplementary Fig. S23).

### Zeta (ζ) Potential Measurement

Zeta potential values were determined at 25 °C on a Brookhaven ZetaPALS (Brookhaven Instrument, USA). The instrument utilizes phase analysis light scattering to provide an average over multiple particles.

## Additional Information

**How to cite this article**: Zhang, C.-C. *et al.* Size Switchable Supramolecular Nanoparticle Based on Azobenzene Derivative within Anionic Pillar[5]arene. *Sci. Rep.* **6**, 37014; doi: 10.1038/srep37014 (2016).

**Publisher’s note:** Springer Nature remains neutral with regard to jurisdictional claims in published maps and institutional affiliations.

## Supplementary Material

Supplementary Information

## Figures and Tables

**Figure 1:**
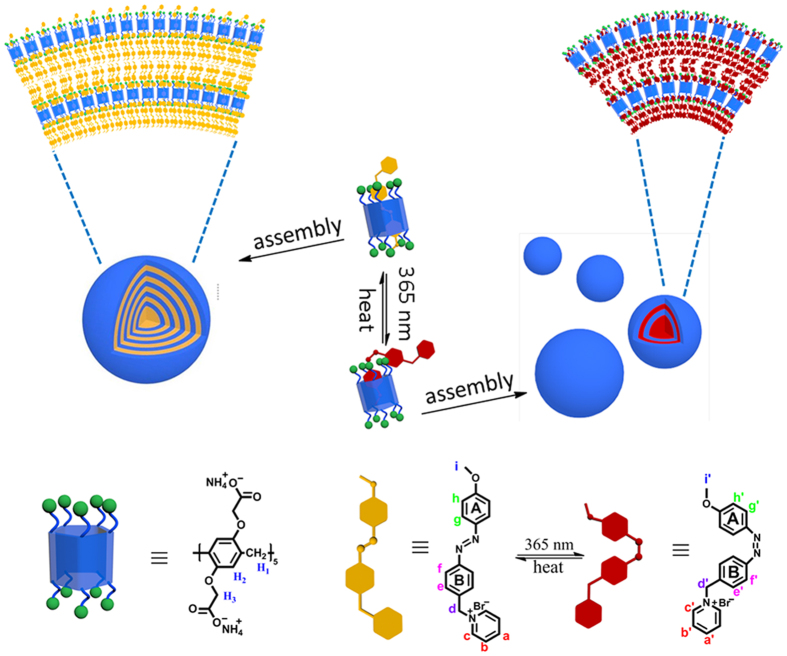
Schematic illustration of the photo/thermal-stimulation size switchable supramolecular assembly between the **2C-WP5A** and **G**.

**Table 1:** Association constant (*K*_a_), standard enthalpic (ΔH°), and entropic changes (TΔS°) for inclusion complex of **2C-WP5A** and **G** in deionized water at 25 °C obtained by ITC.

Guest	*K*_a_(M^−1^)	ΔH°(kJ. mol^−1^)	TΔS(kJ. mol^−1^)	ΔG°(kJ. mol^−1^)
*trans-*G	(2.60 ± 0.06) × 10^4^	−44.71 ± 1.11[b]	−19.49 ± 1.15	−25.22 ± 0.04
*mix-*G	(3.19 ± 0.02) × 10^4^	−39.68 ± 0.34	−13.97 ± 0.32	−25.71 ± 0.23
*cis-*G[a]	(3.25 ± 0.10) × 10^4^	−39.12 ± 0.50	−13.35 ± 0.48	−25.77 ± 0.25

^[a]^The *K*_a_ value and thermodynamic parameters of ***cis-*****G** were calculated by the follow equation: ***cis-*****G**_*K*a value or thermodynamic parameters_ = (***mix-*****G**_*K*a value or thermodynamic parameters_ **−** 10% ***trans*****-G**_*K*a value or thermodynamic parameters_)/90%.

^[b]^The standard error.
