# Supplementary material for: Size Switchable Supramolecular Nanoparticle Based on Azobenzene Derivative within Anionic Pillar[5]arene
Source: Sci Rep. 2016 Nov 16;6:37014. doi: 10.1038/srep37014 (PMC5111115; doi:10.1038/srep37014)
Supplement: Supplementary Information [file srep37014-s1.doc]

**Supporting Information for**

**Size Switchable Supramolecular Nanoparticle Based on Azobenzene Derivative within Anionic Pillar[5]arene**

Cai-Cai Zhang^1^, Sheng-Hua Li^1,2^, Cui-Fang Zhang^1^, and Yu Liu^1,2^*

^1^Department of Chemistry, State Key Laboratory of Elemento-Organic Chemistry, Nankai University, Tianjin 300071, P. R. China.

^2^Collaborative Innovation Center of Chemical Science and Engineering (Tianjin), Nankai University, Tianjin 300071, P. R. China

*Address correspondence to yuliu@nankai.edu.cn

**Table of Contents**

[1. Synthesis procedures 3](#__RefHeading___Toc463780490)

[2. ^1^H NMR spectra, ^13^C NMR spectra and HRMS spectra of compound **B, C, G** and **G′.** 5](#__RefHeading___Toc463780491)

[3. The Job’s plot of **2C-WP5A*trans*-G** 10](#__RefHeading___Toc463780492)

[4. Complexation-induced chemical shifts of the protons of ***trans*-G** in the presence of **2C-WP5A** 11](#__RefHeading___Toc463780493)

[6. Host-guest complexation for **2C-WP5A** and ***trans-*G′** 13](#__RefHeading___Toc463780494)

[7. Association constant determination for the complexation between **2C-WP5A** and ***trans-*G′** 13](#__RefHeading___Toc463780495)

[8. Photoresponsive behavior of **G** 15](#__RefHeading___Toc463780496)

[9. Control experiments to HIA 16](#__RefHeading___Toc463780497)

[10. Zeta potential of **2C-WP5A＋G** assembly 17](#__RefHeading___Toc463780498)

[11. Repetitiveness of the size switching of the supramolecular assembly 18](#__RefHeading___Toc463780499)

[12. Isothermal titration calorimetry (ITC) 19](#__RefHeading___Toc463780500)

[13. Supplementary information references 19](#__RefHeading___Toc463780501)

# 1. Synthesis procedures


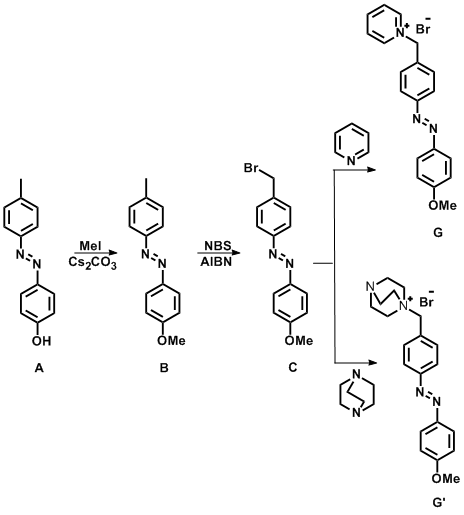


**Supplementary Figure S1.** Synthetic routes of compounds **B**, **C**, **G** and **G′**.

**The synthesis of compound B:**

Compound **A** was synthesized according to the literature^1^.

Compound **A** (2.12 g, 10 mmol) and Cs_2_CO_3_ (4.89 g, 15 mmol) were dissolved/suspended in acetonitrile (100 mL), then iodomethane (7.09 g, 50 mmol) was added, and the mixture was stirred for 2 days at room temperature. The solution was concentrated under reduced pressure to remove excess iodomethane and the residue was dissolved in CH_2_Cl_2_. The resultant solution was washed with H_2_O. The organic phase was collected, dried over anhydrous Na_2_SO_4_ and concentrated to give a yellow product (2.14g, 95%) ^1^H NMR (400 MHz, CDCl_3_) δ 7.90 (d, *J* = 8.9 Hz, 2H), 7.79 (d, *J* = 8.2 Hz, 2H), 7.30 (d, *J* = 8.1 Hz, 2H), 7.01 (d, *J* = 9.0 Hz, 2H), 3.89 (s, 3H), 2.43 (s, 3H). The data is in accord with the reported in the literature^2^.

**The synthesis of** **compound C:**

Compound **B** (2.14 g, 9.45 mmol) and N-bromosuccinimide (1.68 g, 9.45 mmol) were dissolved in CCl_4_ (35 mL) under a nitrogen atmosphere. Then azobisisobutyronitrile (58.96 mg, 0.35 mmol) was added, and the mixture was heated at 77°C for 24h. After cooling down to room temperature, the solution was concentrated under reduced pressure. The residue was separated by column chromatography on silica gel (petroleum ether /ethyl acetate = 50:1, *v/v*) to get an orange solid (2.82g, 98%).^1^H NMR (400 MHz, CDCl_3_) δ 7.92 (d, *J* = 8.9 Hz, 2H), 7.85 (d, *J* = 8.3 Hz, 2H), 7.52 (d, *J* = 8.3 Hz, 2H), 7.02 (d, *J* = 8.9 Hz, 2H), 4.56 (s, 2H), 3.90 (s, 3H). ^13^C NMR (100 MHz, CDCl3) δ 162.31, 152.55, 147.04, 139.86, 129.90, 125.02, 122.92, 114.30, 55.66, 33.00. HRMS for C_14_H_13_BrN_2_O: calcd. [M+H]^+^: 305.0289/307.0269, found: 305.0286/307.0267.

**The synthesis of compound G:**

Compound **C** (2.82 g, 9.24 mmol) and pyridine (2 mL, 29.84 mmol) were dissolved in 25 ml acetonitrile, 5 mL dichloromethane was added. After stirred for 4d at room temperature, the reaction mixture was poured into 50 mL diethyl ether, the precipitate was collected by filtration, washed with diethyl ether for several times, dried overnight in a vacuum to give product **G** as orange solid (2.84 g, 80%). ^1^H NMR (400 MHz, D_2_O) δ 8.93 (d, *J* = 5.4 Hz, 2H), 8.56 (t, *J* = 7.6 Hz, 1H), 8.07 (t, *J* = 6.4 Hz, 2H), 7.85 (d, *J* = 8.5 Hz, 2H), 7.81 (d, *J* = 8.2 Hz, 2H), 7.59 (d, *J* = 7.6 Hz, 2H), 7.11 (d, *J* = 8.0 Hz, 2H), 5.88 (s, 2H), 3.88 (s, 3H). ^13^C NMR (100MHz, D_2_O) δ 161.69, 152.07, 146.18, 145.63, 144.07, 134.78, 129.84, 128.49, 124.57, 122.98, 113.98, 63.66, 55.32. HRMS for C_19_H_18_BrN_3_O: calcd. [M–Br]^+^: 304.1450, found: 304.1447.

**The synthesis of compound G′:**

The synthesis procedure of **G′** is similar to **G**.

^1^H NMR (400 MHz, D_2_O) δ 7.93 (d, *J* = 8.6 Hz, 2H), 7.90 (d, *J* = 8.5 Hz, 2H), 7.67 (d, *J* = 8.3 Hz, 2H), 7.17 (d, *J* = 9.0 Hz, 2H), 4.57 (s, 2H), 3.92 (s, 3H), 3.51–3.44 (m, 6H), 3.20–3.13 (m, 6H). ^13^C NMR (100MHz, D_2_O) δ 162.49, 153.42, 146.36, 134.19, 128.21, 124.92, 122.50, 114.77, 67.66, 55.67, 52.03, 44.18. HRMS for C_20_H_25_BrN_4_O: calcd. [M–Br]^+^: 337.2028, found: 337.2035.

# 2. ^1^H NMR spectra, ^13^C NMR spectra and HRMS spectra of compound B, C, G and G′.


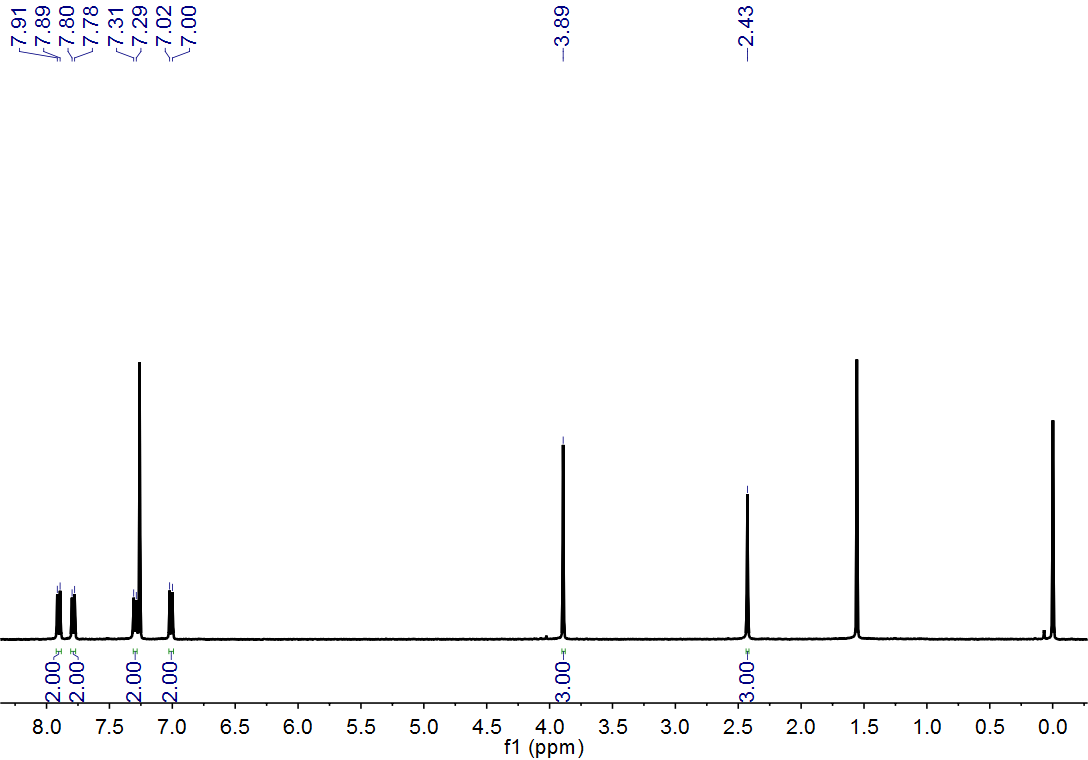


**Supplementary Figure S2.** ^1^H NMR spectrum (400 MHz, CDCl_3_, 25 °C) of **B**.


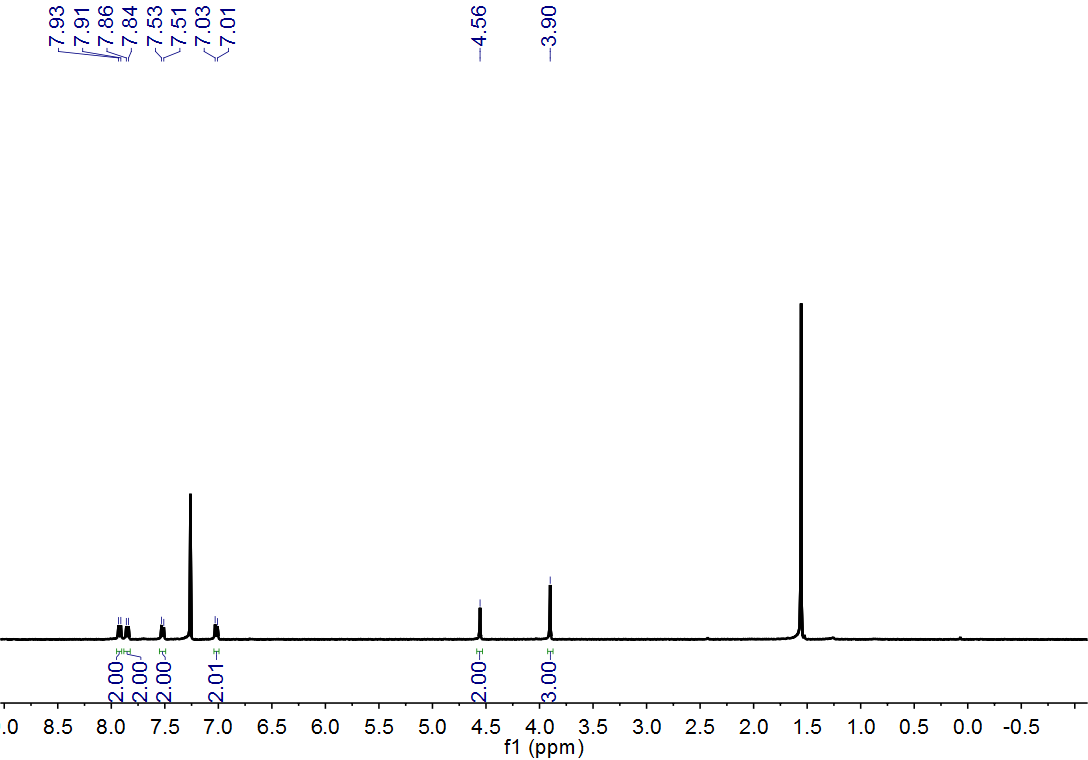


**Supplementary Figure S3.** ^1^H NMR spectrum (400 MHz, CDCl_3_, 25 °C) of **C**.


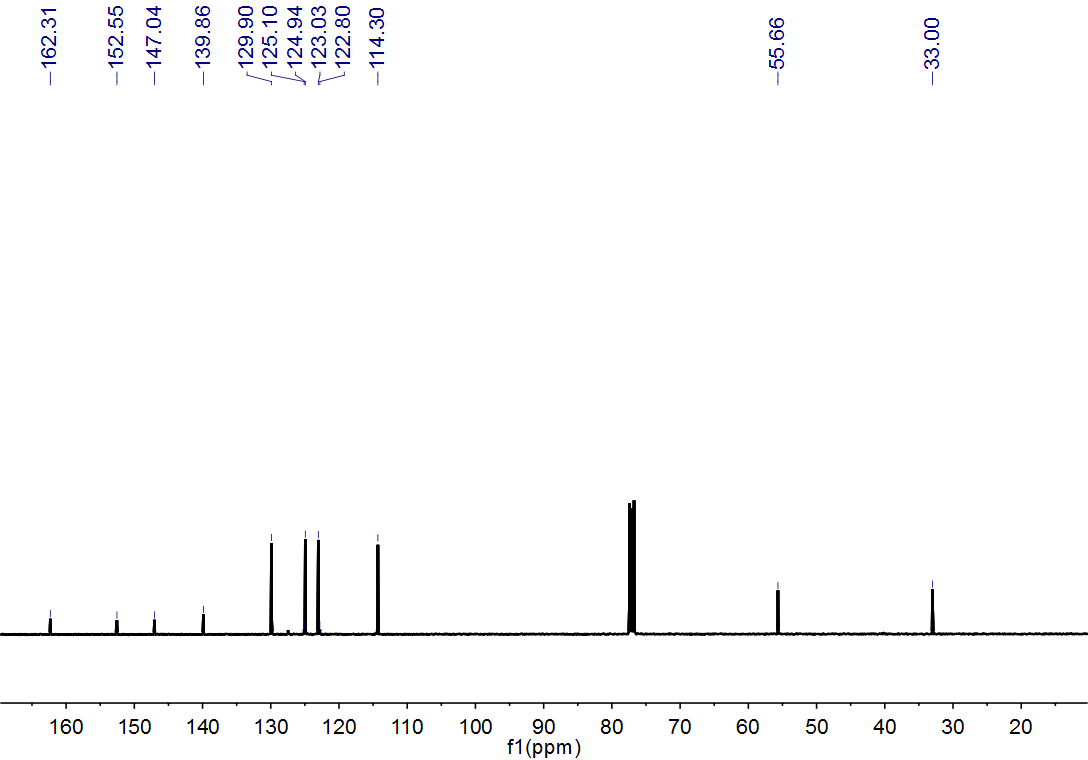


**Supplementary Figure S4.** ^13^C NMR spectrum (100 MHz, CDCl_3_, 25 °C) of **C**.


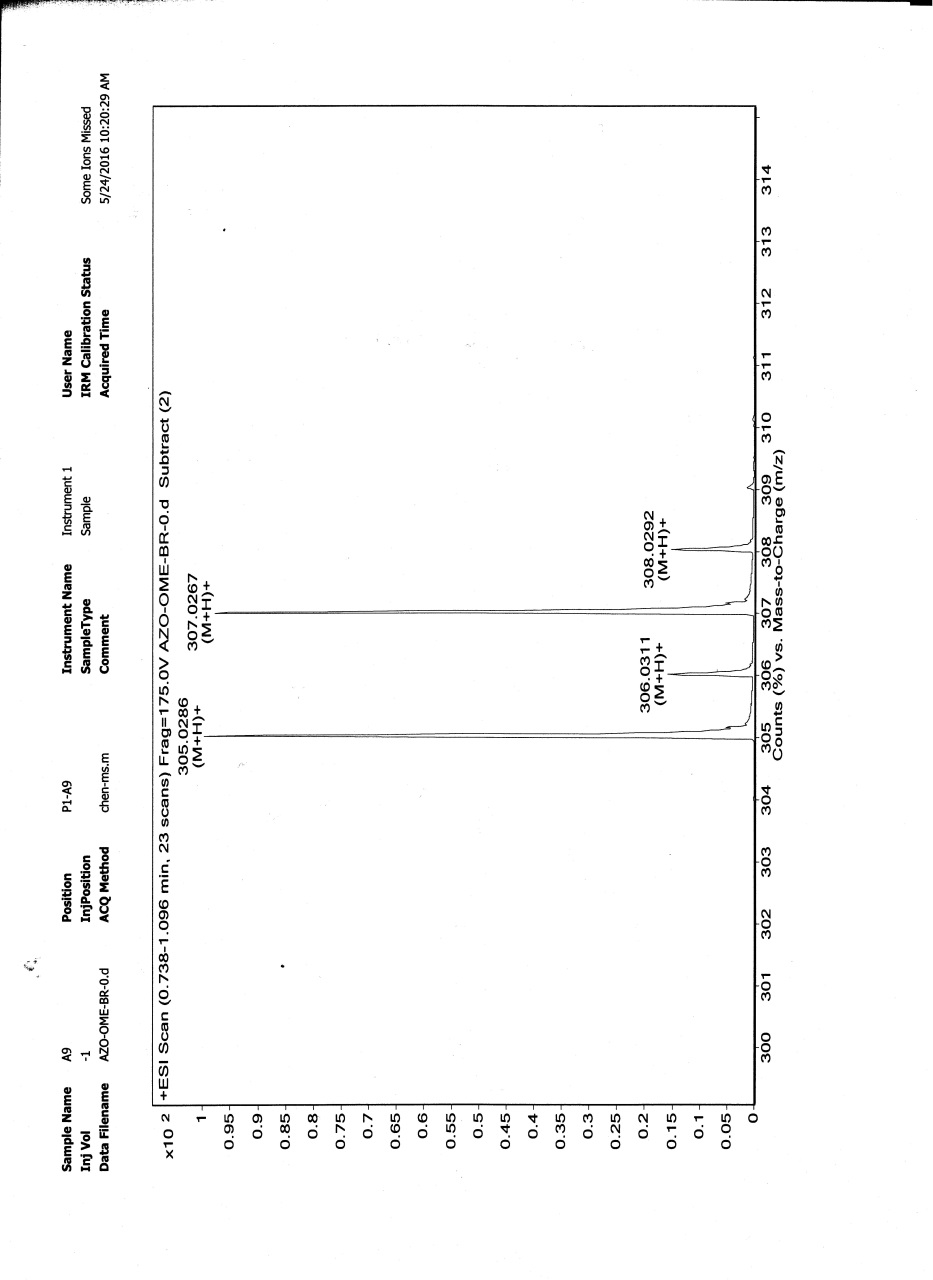


**Supplementary Figure S5.** HRMS spectrum of **C** (C_14_H_13_BrN_2_O). The peak at *m*/*z* 305.0286/307.0267 is assigned to [M+H]^+^, calcd.: 305.0289/307.0269.


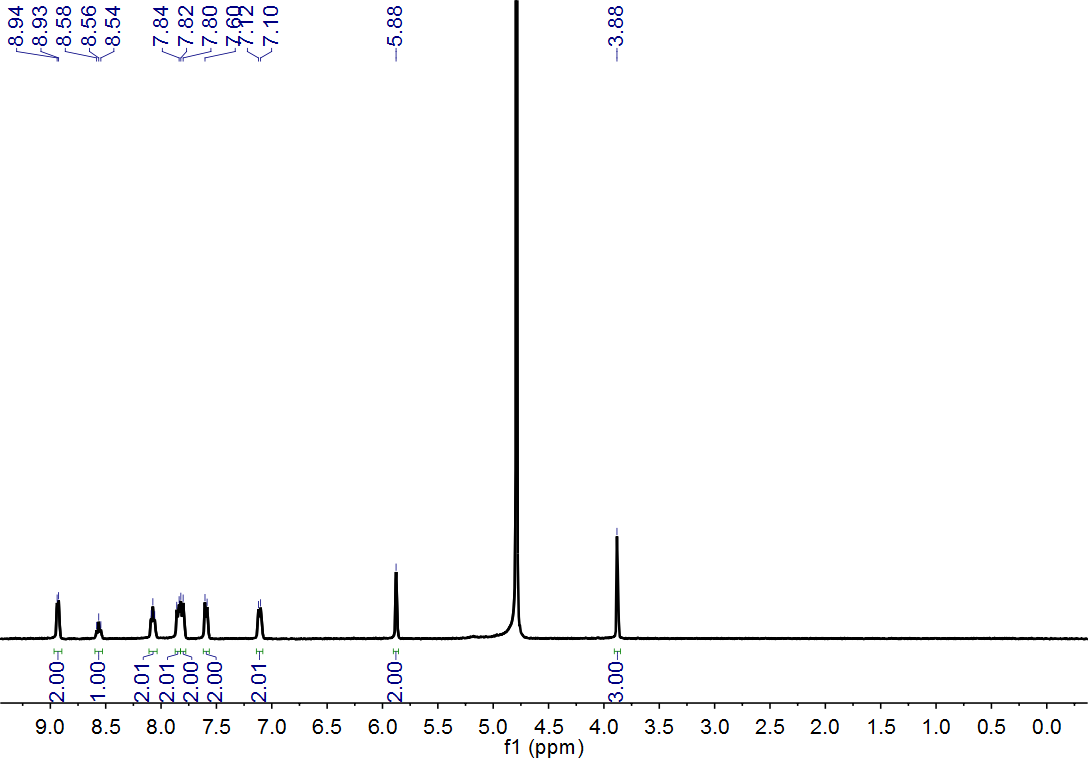


**Supplementary Figure S6.** ^1^H NMR spectrum (400 MHz, CDCl_3_, 25 °C) of **G**.


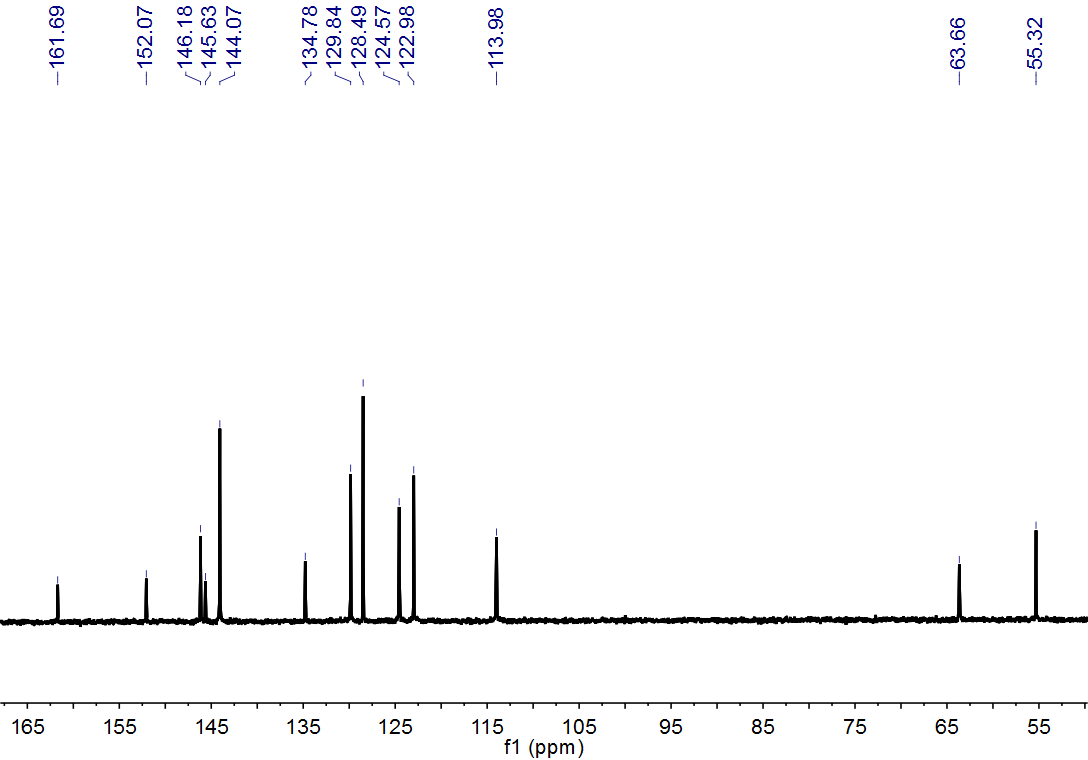


**Supplementary Figure S7.** ^13^C NMR spectrum (100 MHz, CDCl_3_, 25 °C) of **G.**


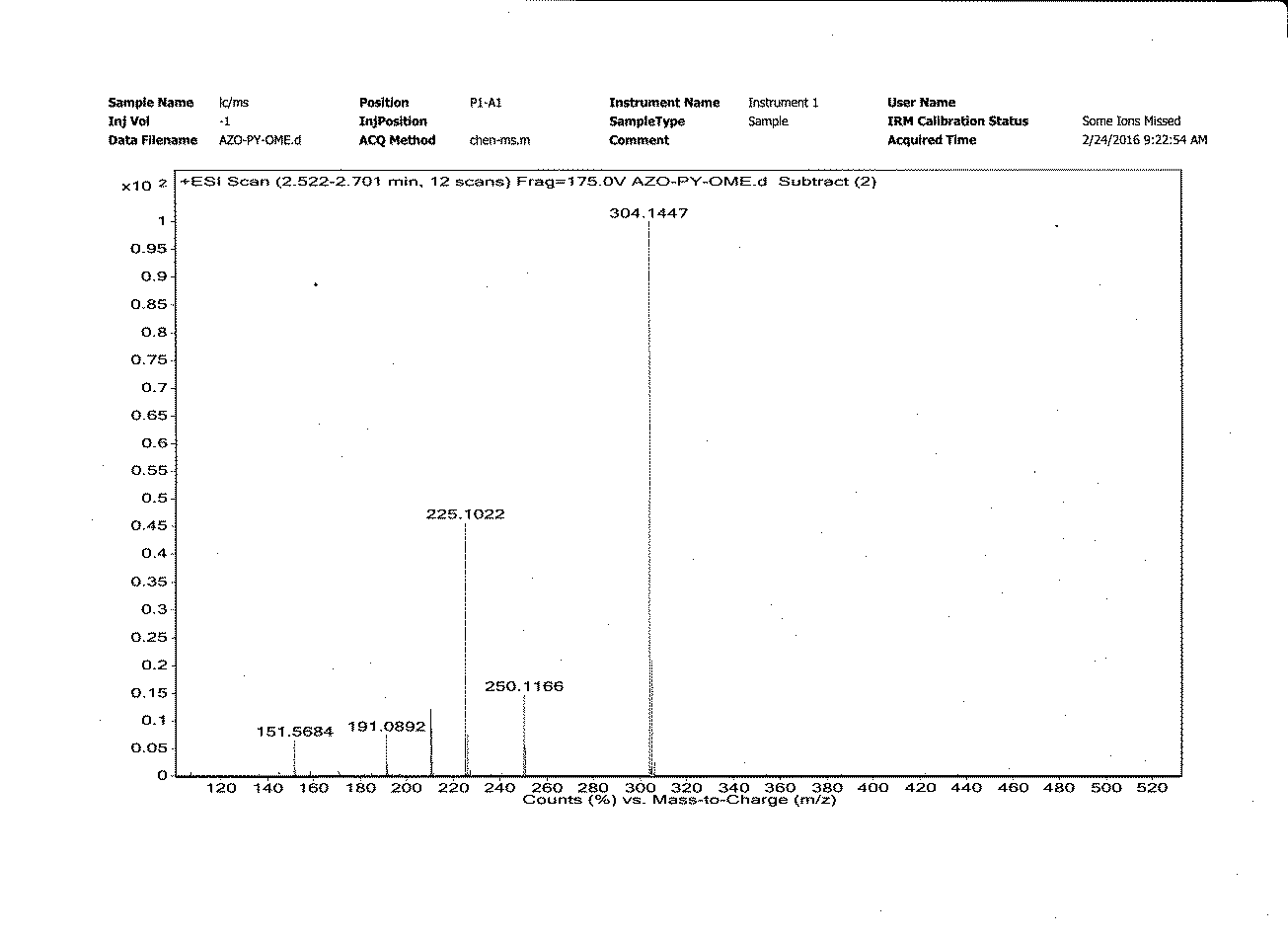
**Supplementary Figure S8.** HRMS spectrum of **G** (C_19_H_18_BrN_3_O). The peak at *m*/*z* 304.1447 is assigned to [M–Br]^+^, calcd.: 304.1450


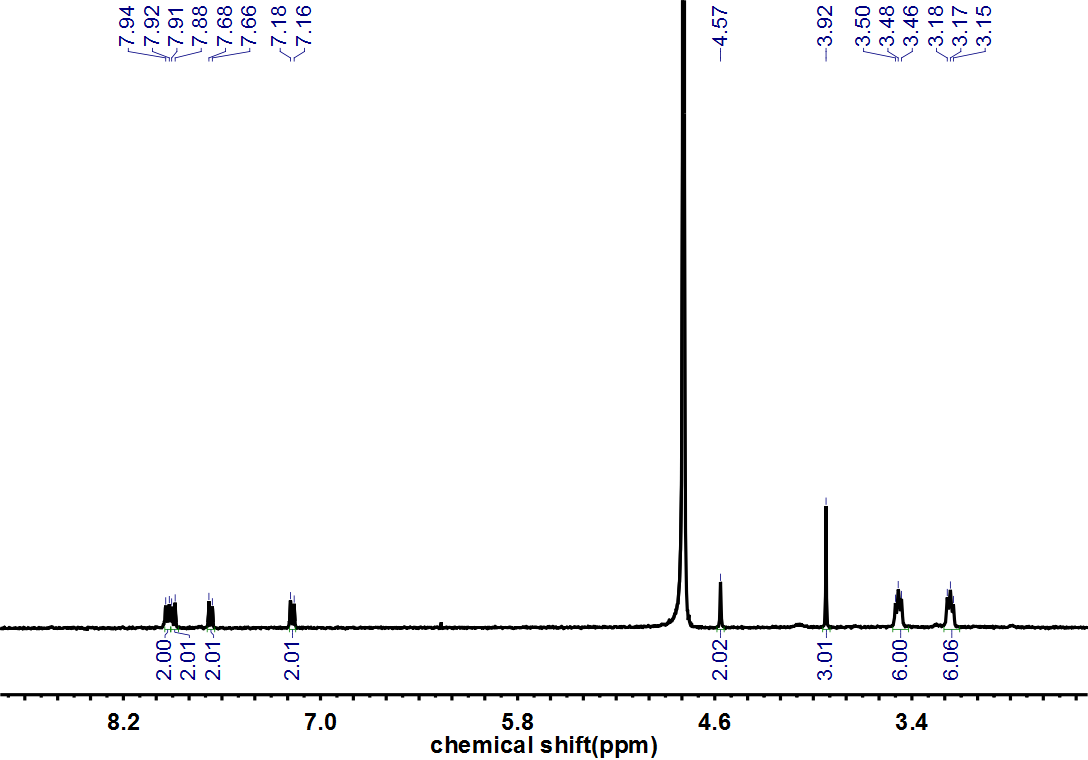
**Supplementary Figure S9.** ^1^H NMR spectrum (400 MHz, CDCl_3_, 25 °C) of **G′**


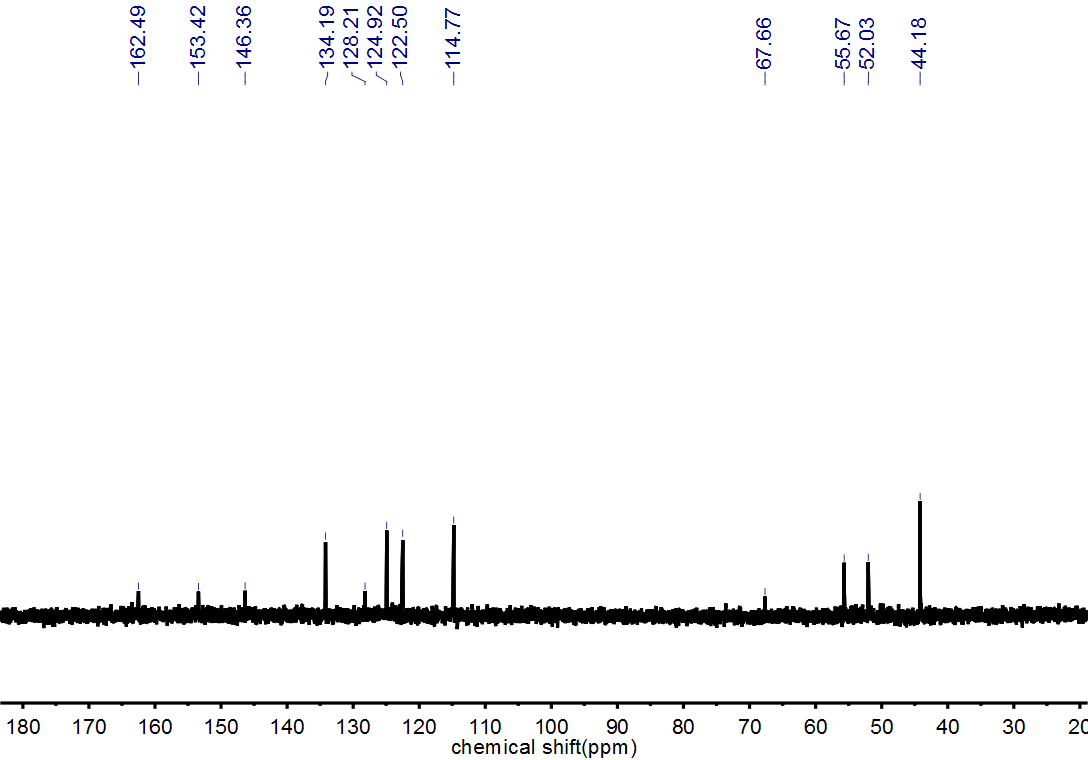


**Supplementary Figure S10.** ^13^C NMR spectrum (100 MHz, CDCl_3_, 25 °C) of **G′.**

**
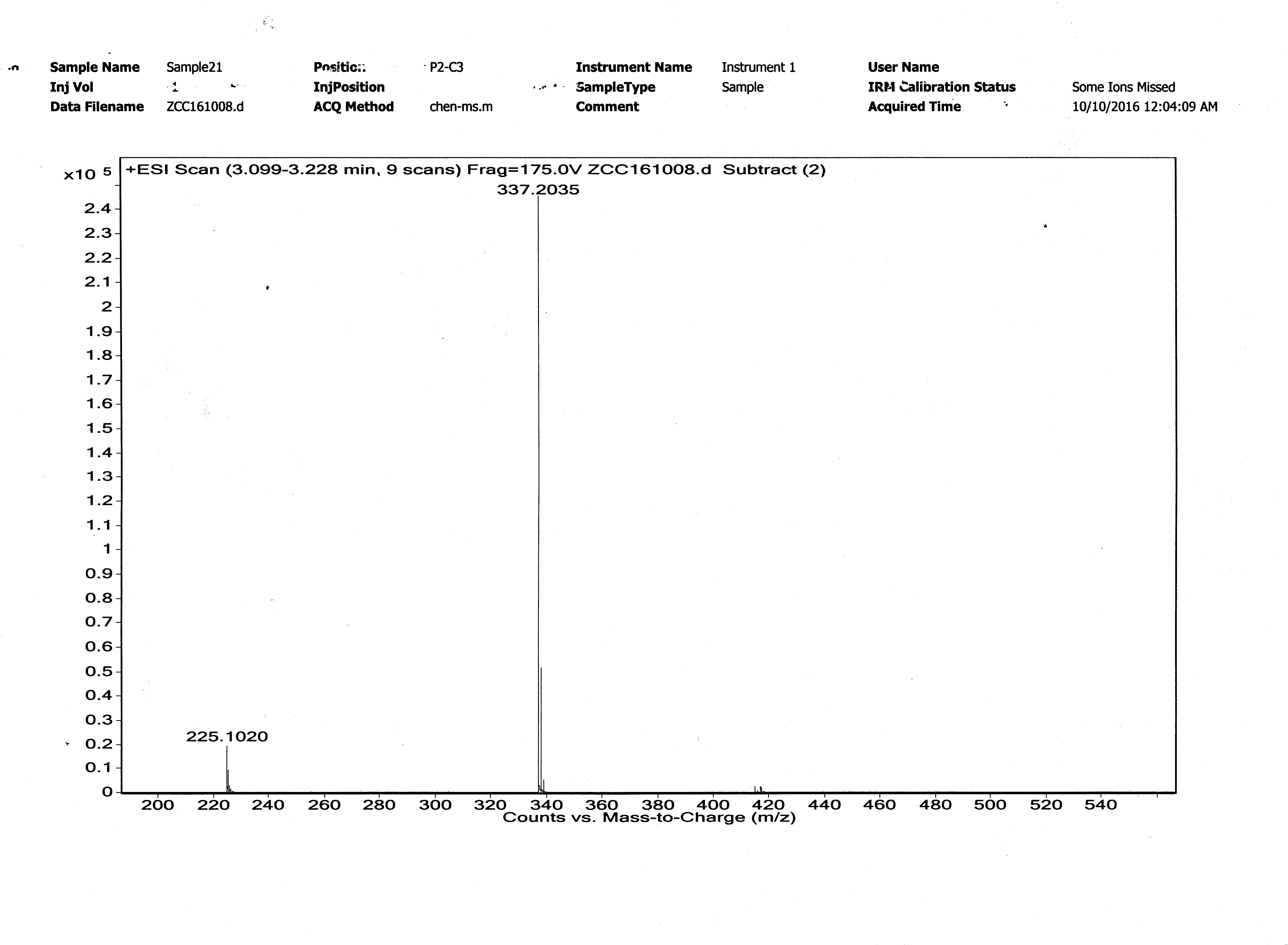
**

**Supplementary Figure S11.** HRMS spectrum of **G2** (C_20_H_25_BrN_4_O). The peak at *m*/*z* 337.2035 is assigned to [M–Br]^+^, calcd.: 337.2028

# 3. The Job’s plot of 2C-WP5A*trans*-G1


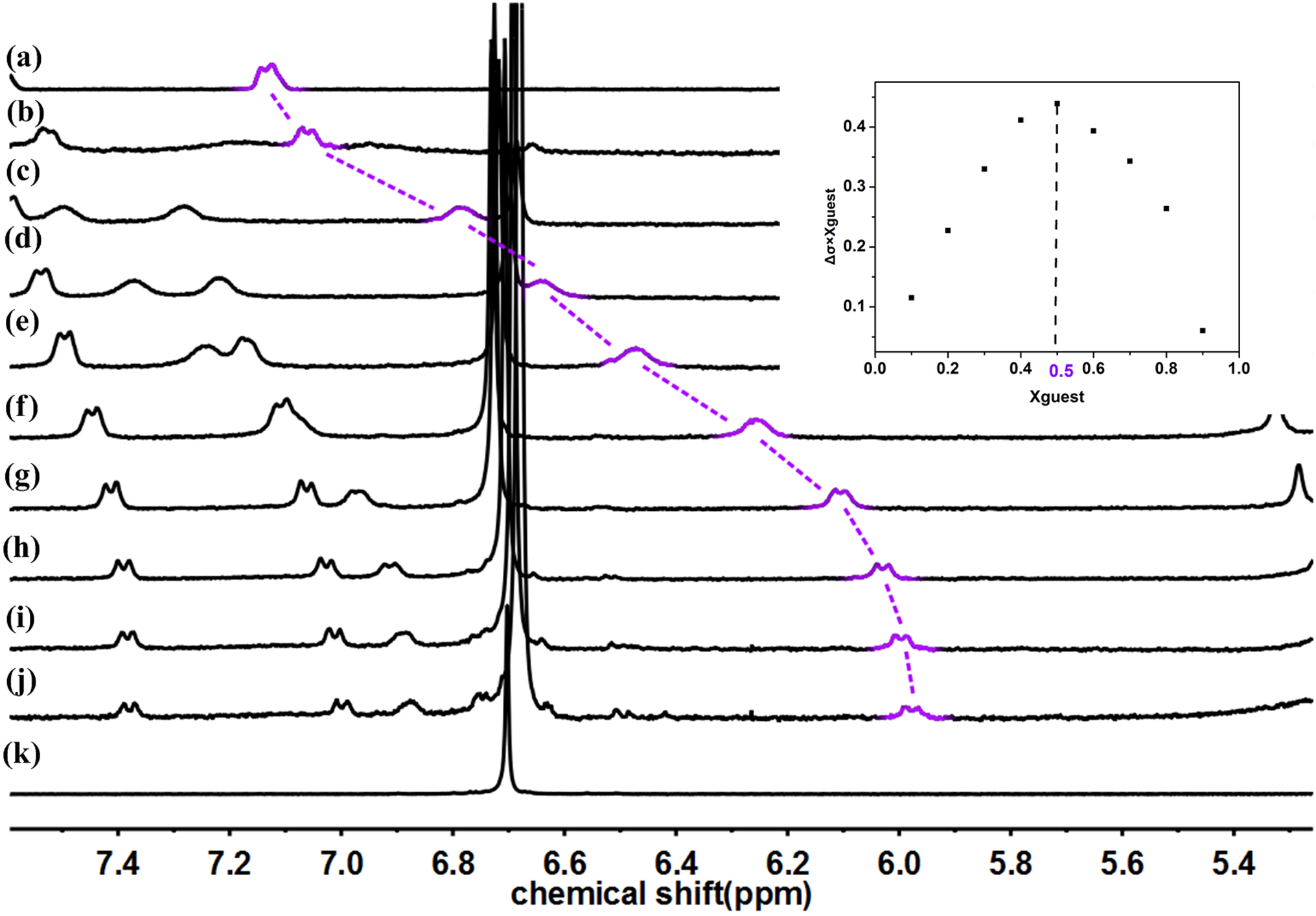


**Supplementary Figure S12.** ^1^H NMR spectra ([**2C-WP5A**] + [***trans*-G**] =5 mM, D_2_O, 25 °C, 400 MHz) of the chemical shift changes of H_h_ on ***trans*-G** with the molar ratio of ***trans*-G** is: (a) individual ***trans*-G**, (b) 0.9, (c) 0.8, (d) 0.7, (e) 0.6, (f) 0.5, (g) 0.4, (h) 0.3, (i) 0.2, (j) 0.1, and (k) individual **2C-WP5A**. Insert: Job’s plot showing the 1:1 stoichiometry of the complex between **2C-WP5A** and ***trans*-G** by ^1^H NMR titration.

# 4. Complexation-induced chemical shifts of the protons of *trans*-G in the presence of 2C-WP5A


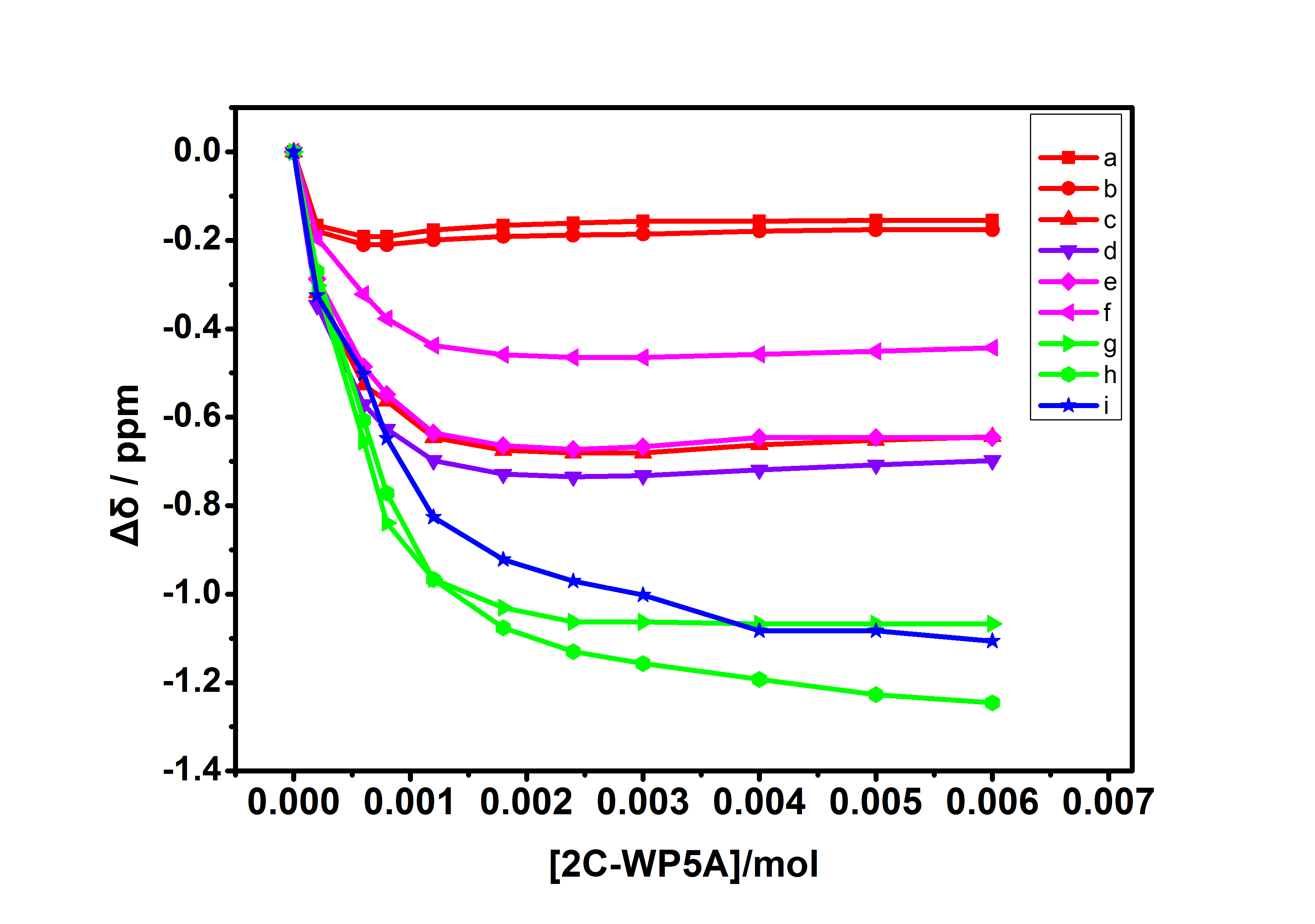
**Supplementary Figure S13.** Complexation-induced chemical shifts (Δδ, ppm, D_2_O, 25°C, 400MHz) of the protons of ***trans*-G** (1 mM) in the presence of various concentrations of **2C-WP5A**.

1. **The 2D ROESY NMR spectrum of 2C-WP5A*trans*-G**


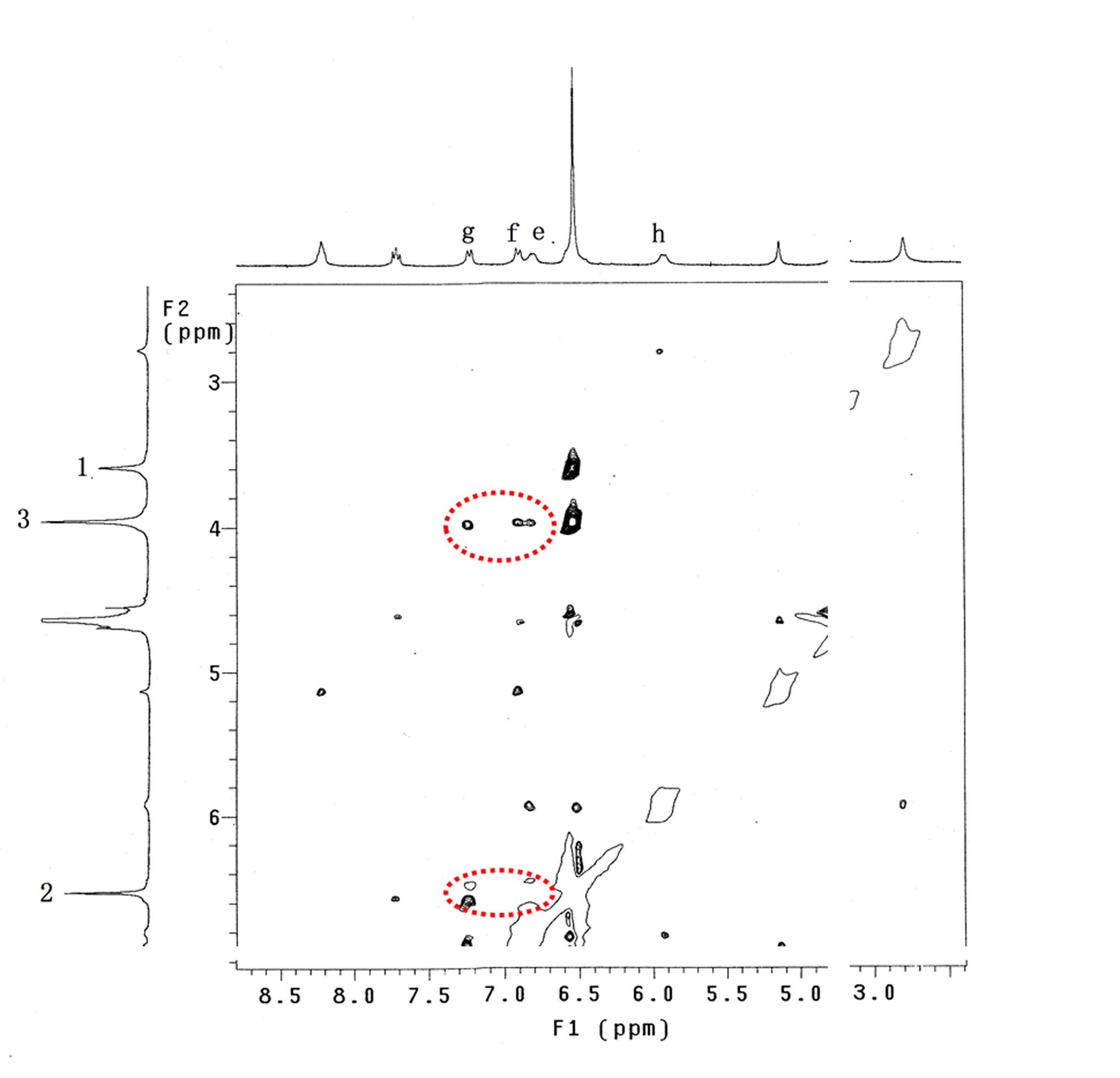


**Supplementary Figure S14.** 2D ROESY NMR spectrum (D_2_O, 25 °C, 300 MHz) of an equimolar mixture ***trans-*G** and **2C-WP5A** (5.0 mM).

# Host-Guest complexation for 2C-WP5A and *trans-*G′


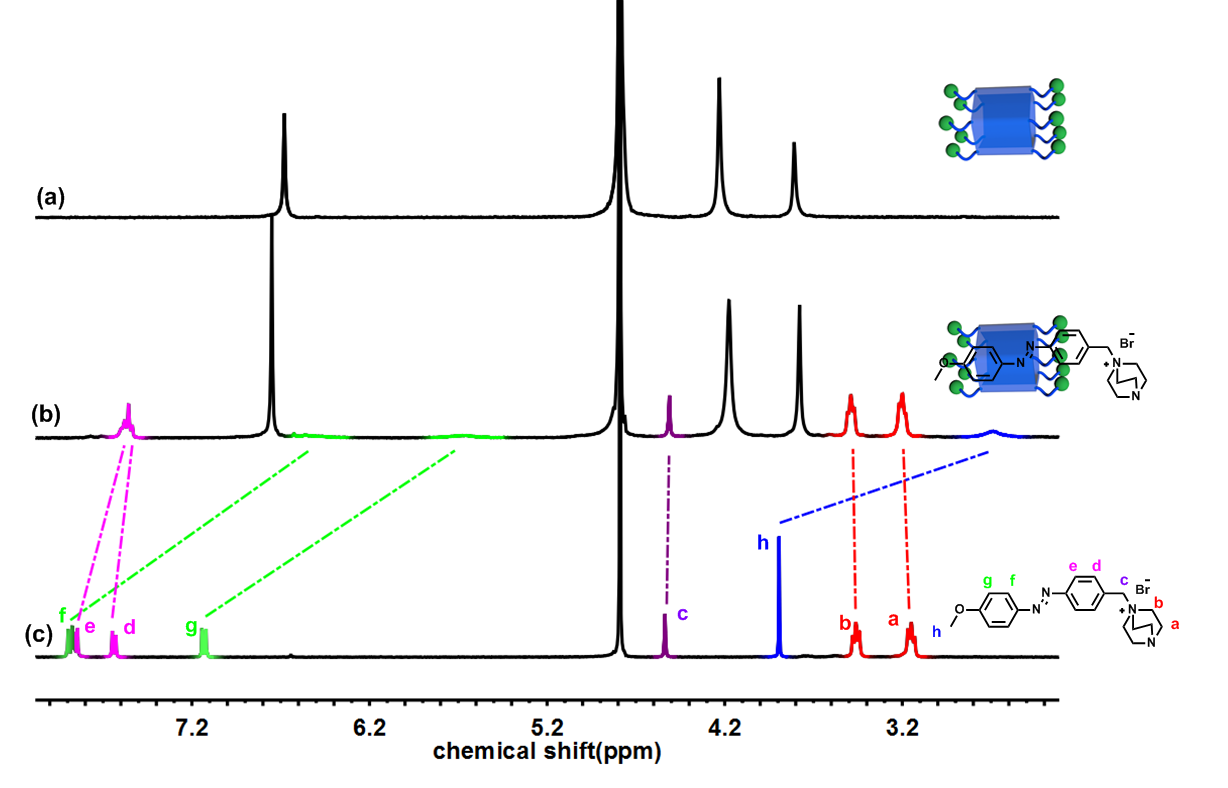


**Supplementary Figure S15.** Partial ^1^H NMR spectra (D_2_O, 25 °C, 400 MHz) of (a) **2C-WP5A** (2.5 mM); (b) ***trans*-G′** (2.5 mM) and **2C-WP5A** (2.5 mM); (c) ***trans-*G′** (2.5 mM).

# Association constant determination for the complexation between 2C-WP5A and *trans-*G′

We try to find out the association constant between **2C-WP5A** and ***trans-*G2** by UV/vis titration using a nonlinear curve fitting ^S3^. The association constants for **2C-WP5A *trans-*G2** is (2.98±0.50)×10^4^ M^-1^,


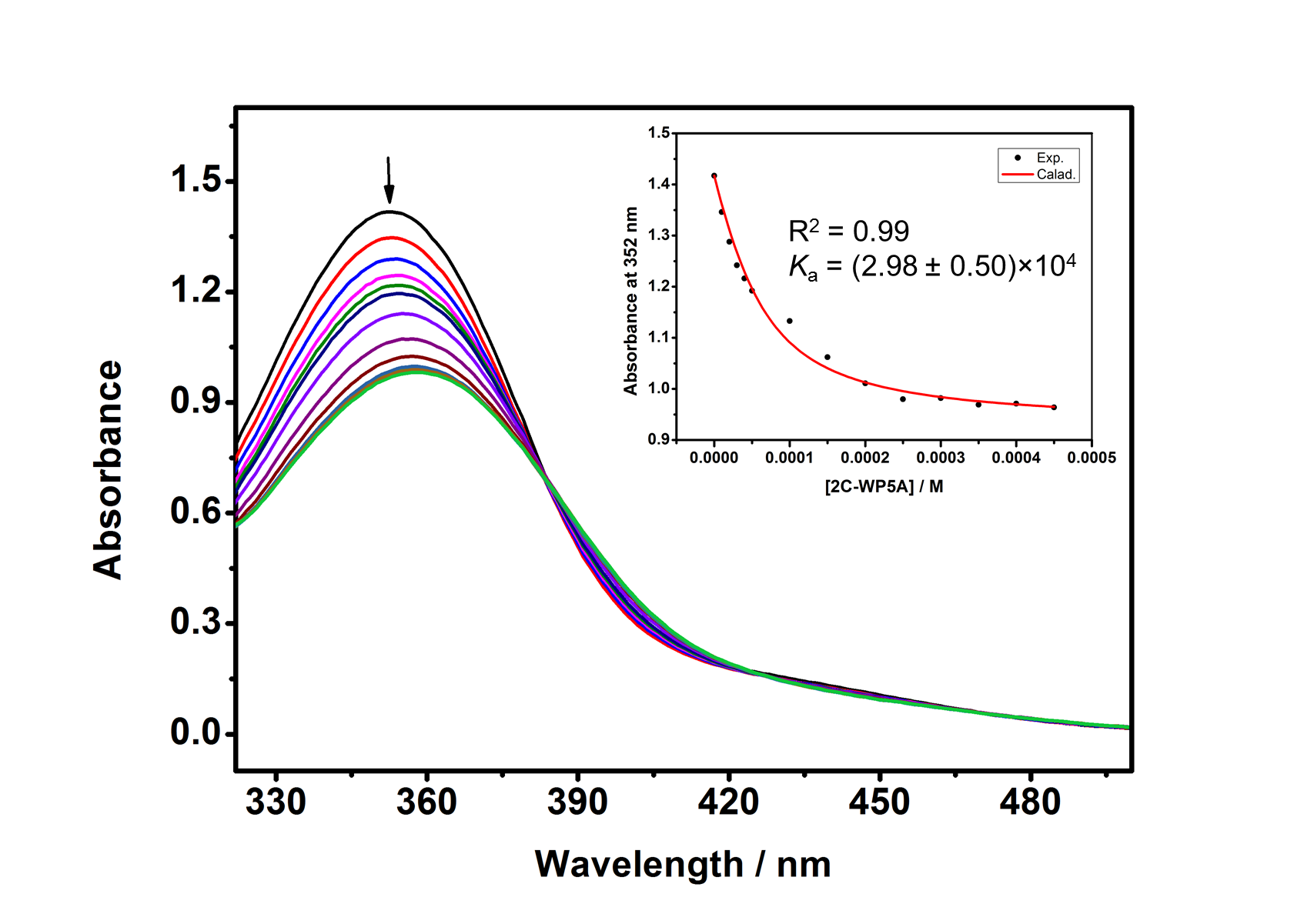


**Supplementary Figure S16.** Alterations of the UV/vis absorbance spectra of ***trans-*G′** (0.05 mM) upon addition of **2C-WP5A** (0−0.45 mM, in aqueous solution at 25 °C) Inset: the nonlinear curve fitting of the variation of UV/vis absorbance intensity with the concentration of **2C-WP5A** to calculate the association constant.

#
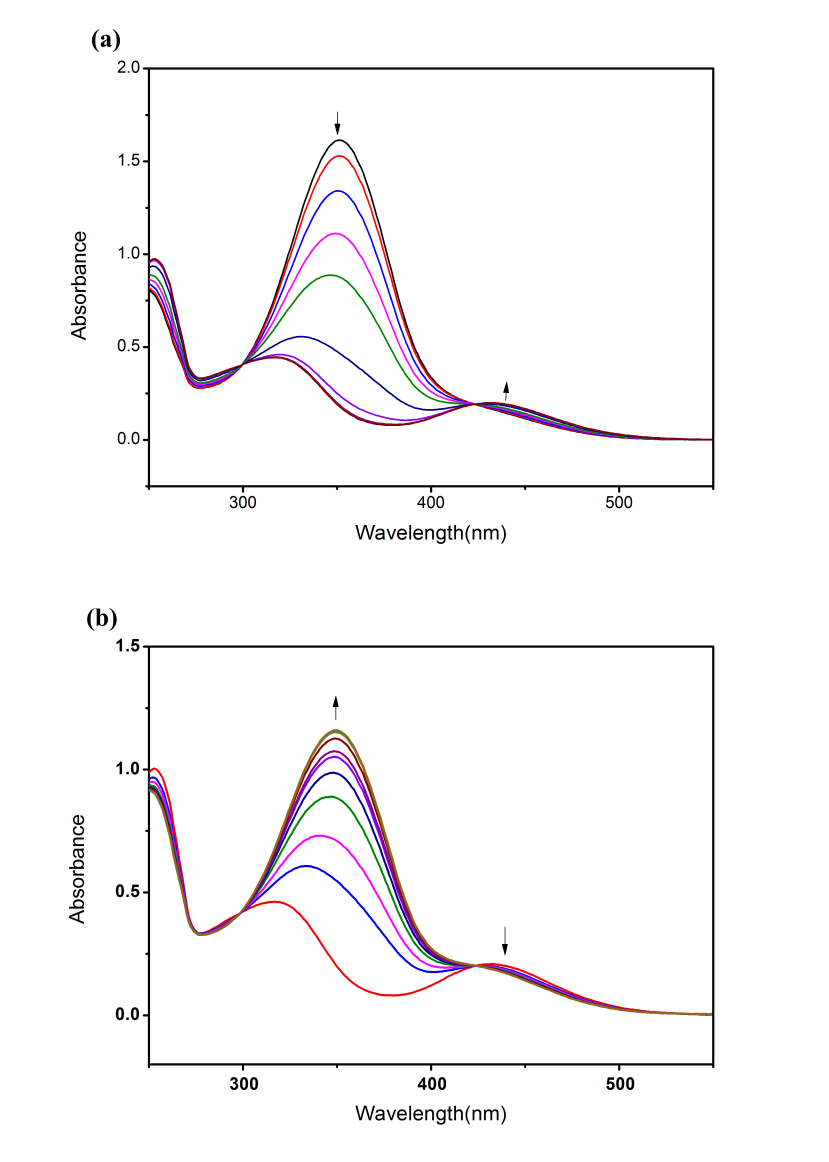
8. Photoresponsive behavior of G

**Supplementary Figure S17.** UV-Vis absorption spectra of an aqueous solution of ***trans-*G** (0.5mM) (a) under UV irradiation at 365 nm for 0s, 4s, 11s, 20s, 30s, 50s, 70s, 90s, and 120s, (b) and upon visible irradiation at 450 nm for 2s, 4s, 6s, 8s, 10s, 12s, 15s, 20s, 35s, 60s, and 90s

# 9. Control experiments to HIA


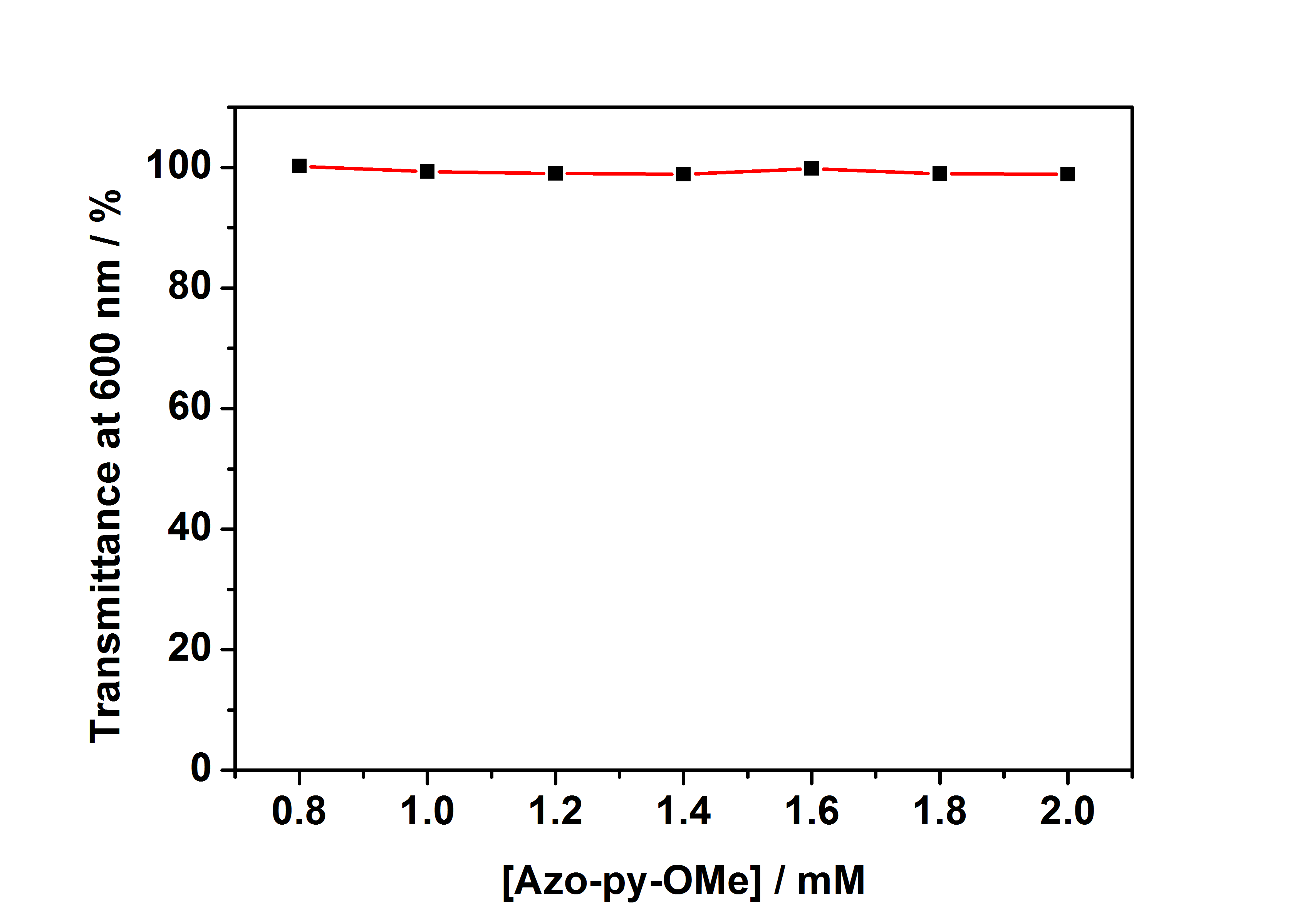


**Supplementary Figure S18.** Dependence of the optical transmittance at 600 nm on ***trans-*G** concentration.


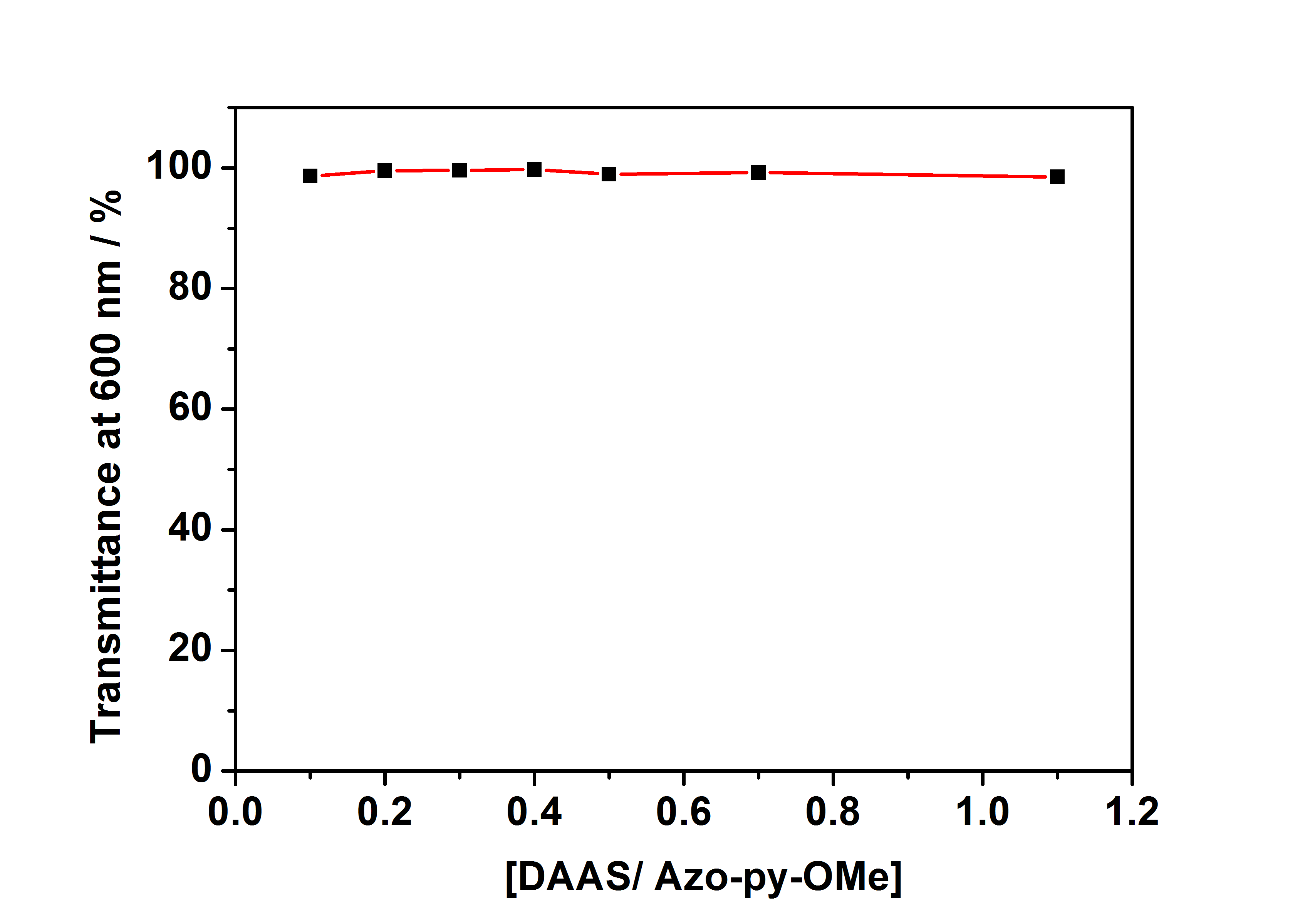


**Supplementary Figure S19.** Optical transmittance of aqueous solutions of **DAAS** at different concentrations in the presence of ***trans-*G** (1.6 mM) at 25 °C. Horizontal axis represents the ratio of **DAAS** to ***trans-*G**.

# 10. Zeta potential of 2C-WP5A＋G assembly


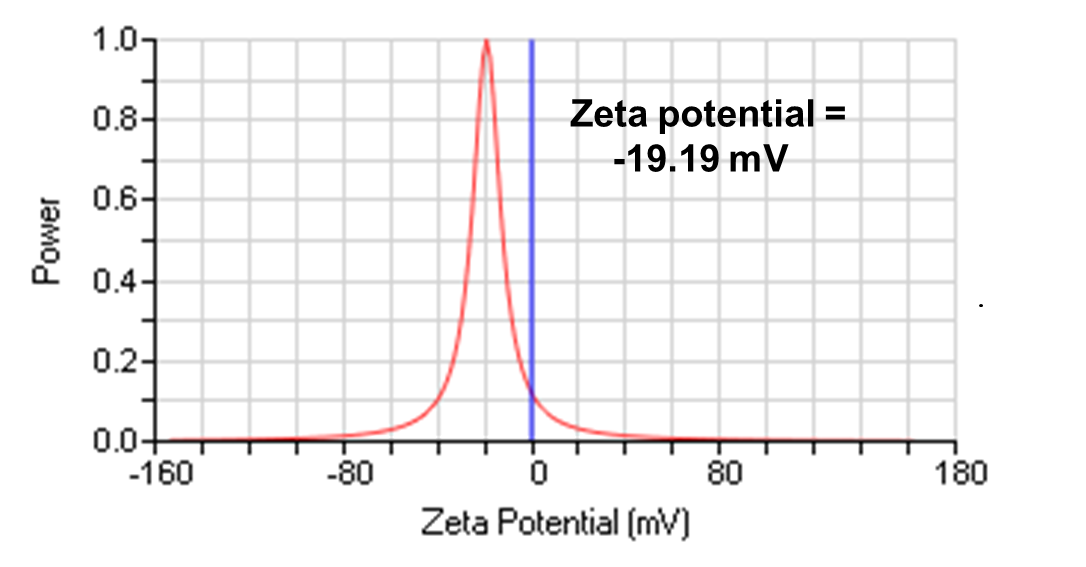


**Supplementary Figure S20.** Zeta potential data of the **2C-WP5A＋*trans-*G** assembly in water at 25 °C.


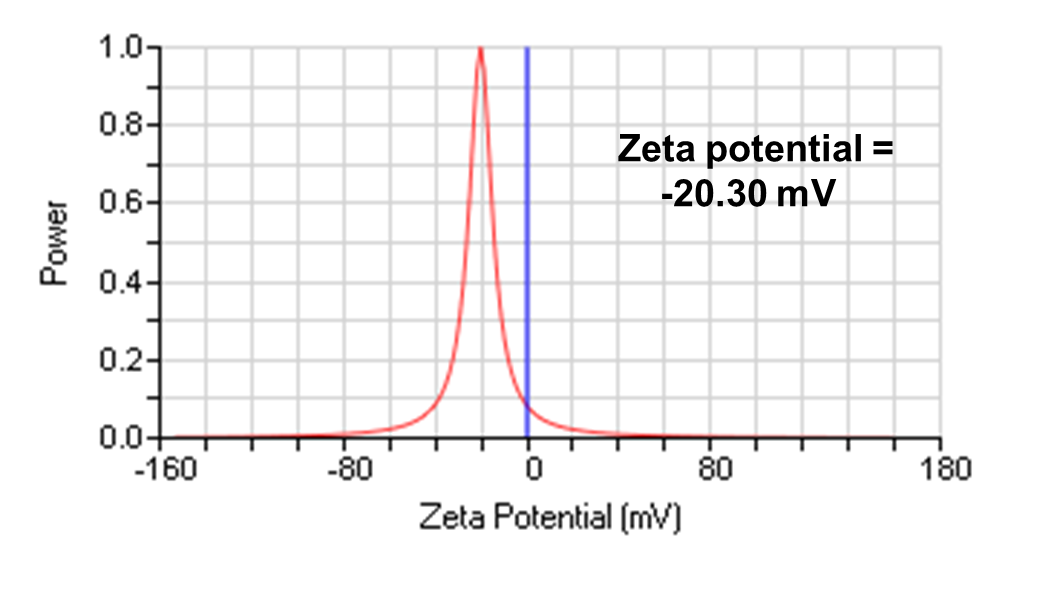


**Supplementary Figure S21.** Zeta potential data of the **2C-WP5A＋*cis-*G** assembly in water at 25 °C.

# 11. Repetitiveness of the size switching of the supramolecular assembly


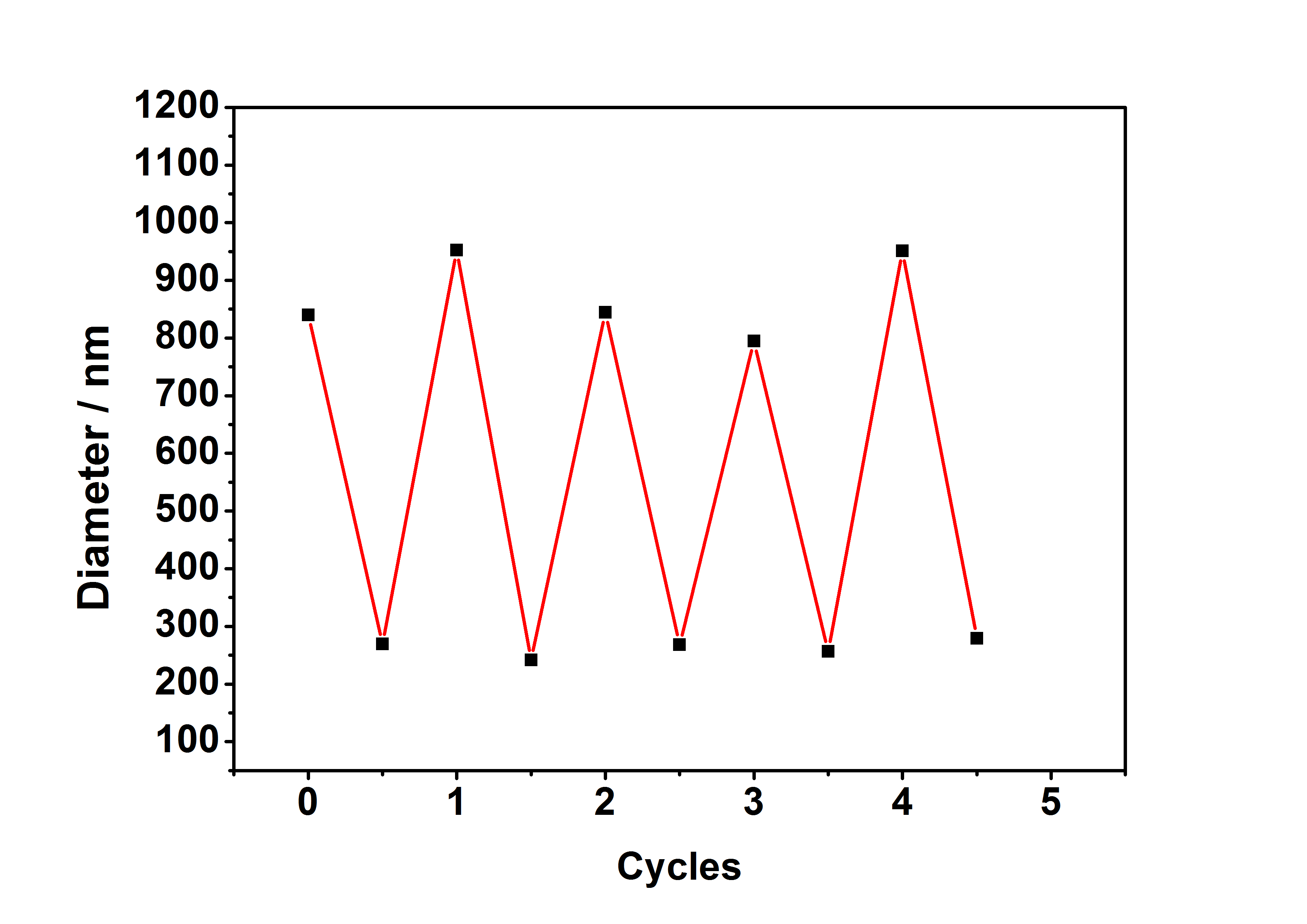


**Supplementary Figure S22.** DLS data of **2C-WP5A＋G** solution observed upon several cycles under irradiation at 365 nm and heating at 80 °C for one hour.

# 12. Isothermal titration calorimetry (ITC)


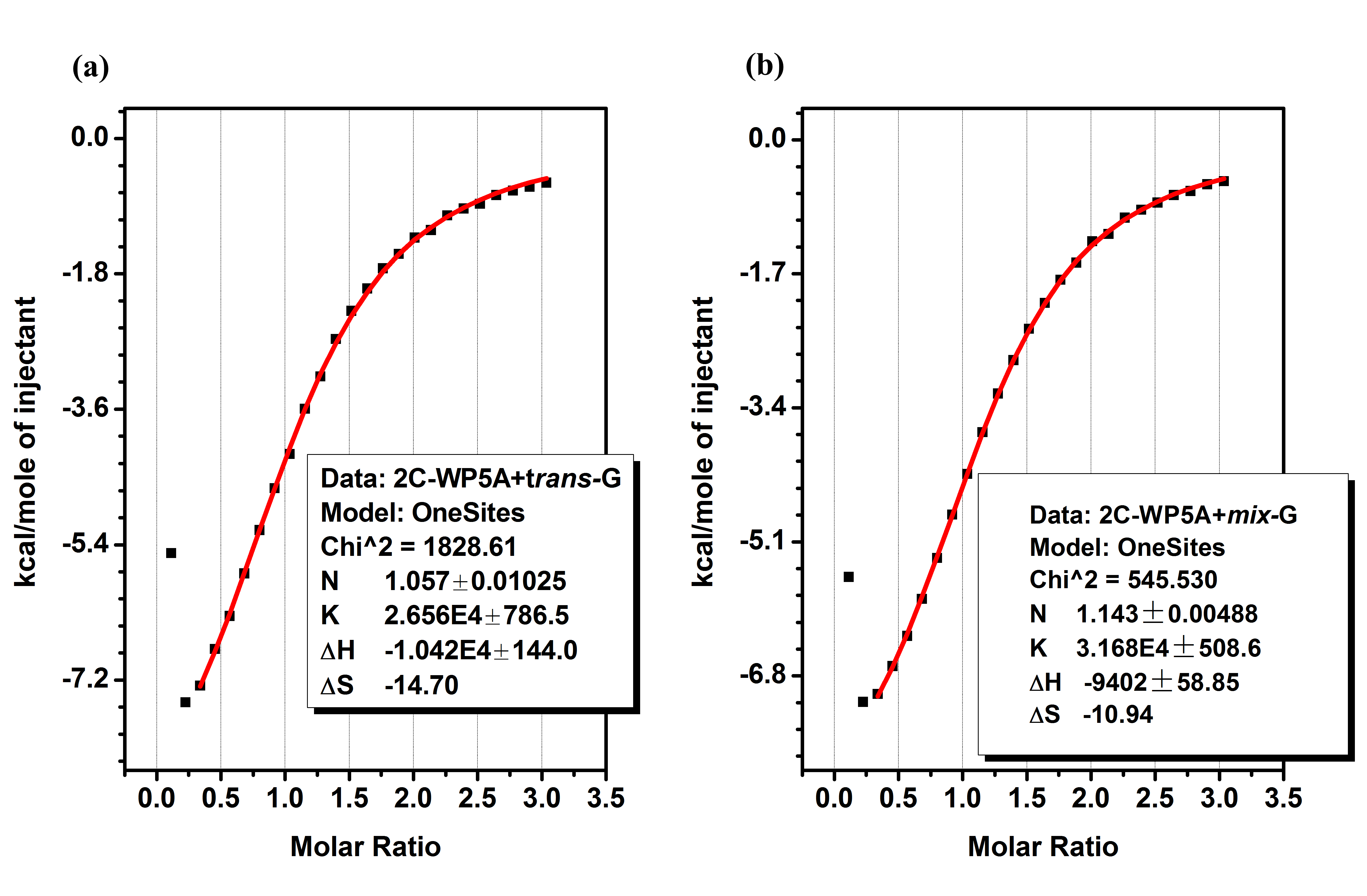


**Supplementary Figure S23.** (a) Microcalorimetric titration of **2C-WP5A** with ***trans-*G** in water at 25 °C; (b) Microcalorimetric titration of **2C-WP5A** with ***trans-*G/c*is-*G** (molar ratio 10/90) in water at 25 °C. “Net” heat effect obtained by subtracting the heat of dilution from the heat of reaction, which was analyzed by computer simulation using the “one set of binding sites” model.

# 13. Supplementary information references

1. Xu, H. & Zeng, X. Synthesis of diaryl-azo derivatives as potential antifungal agents. *Bioorg. Med. Chem. Lett.* **20**, 4193–4195 (2010).

2. Zhang, C.& Jiao, N. Copper-catalyzed aerobic oxidative dehydrogenative coupling of anilines leading to aromatic azo compounds using dioxygen as an oxidant. *Angew. Chem. Int. Ed.* **49**, 6174–6177 (2010).

3. Liu, Y., Li, B., You, C. C., Wada, T. & Inoue, Y. Molecular recognition studies on supramolecular systems. 32.^1^ molecular recognition of dyes by organoselenium-bridged bis(β-cyclodextrin)s. *J. Org. Chem.* **66**, 225–232 (2001).
